# Combined biotic stresses trigger similar transcriptomic responses but contrasting resistance against a chewing herbivore in *Brassica nigra*

**DOI:** 10.1186/s12870-017-1074-7

**Published:** 2017-07-17

**Authors:** Christelle Bonnet, Steve Lassueur, Camille Ponzio, Rieta Gols, Marcel Dicke, Philippe Reymond

**Affiliations:** 1 Department of Plant Molecular Biology, University of Lausanne, Biophore Building, 1015 Lausanne, Switzerland; 2 Laboratory of Entomology, Wageningen University, P.O. Box 16, 6700 AA Wageningen, The Netherlands

**Keywords:** *Brassica nigra*, *Brevicoryne brassicae*, Combined stresses, Herbivory, *Pieris brassicae*, Transcriptome, *Xanthomonas campestris* pv. *raphani (Xcr)*

## Abstract

**Background:**

In nature, plants are frequently exposed to simultaneous biotic stresses that activate distinct and often antagonistic defense signaling pathways. How plants integrate this information and whether they prioritize one stress over the other is not well understood.

**Results:**

We investigated the transcriptome signature of the wild annual crucifer, *Brassica nigra*, in response to eggs and caterpillars of *Pieris brassicae* butterflies, *Brevicoryne brassicae* aphids and the bacterial phytopathogen *Xanthomonas campestris* pv. *raphani* (*Xcr*). Pretreatment with egg extract, aphids, or *Xcr* had a weak impact on the subsequent transcriptome profile of plants challenged with caterpillars, suggesting that the second stress dominates the transcriptional response. Nevertheless, *P. brassicae* larval performance was strongly affected by egg extract or *Xcr* pretreatment and depended on the site where the initial stress was applied. Although egg extract and *Xcr* pretreatments inhibited insect-induced defense gene expression, suggesting salicylic acid (SA)/jasmonic acid (JA) pathway cross talk, this was not strictly correlated with larval performance.

**Conclusion:**

These results emphasize the need to better integrate plant responses at different levels of biological organization and to consider localized effects in order to predict the consequence of multiple stresses on plant resistance.

**Electronic supplementary material:**

The online version of this article (doi:10.1186/s12870-017-1074-7) contains supplementary material, which is available to authorized users.

## Background

Biotic and abiotic stresses impose a strong pressure on plants in nature. When combined, stresses such as heat, drought or high light intensity have profound effects on crop performance and yields [1]. Plants have developed specific mechanisms to precisely detect environmental changes and respond to complex stress conditions to minimize damage and conserve sufficient resources for growth and reproduction. Over the years, research has focused mainly on responses to a single stress in several model plants including Arabidopsis [2–5]. However, there is a growing recognition for the need to consider the effects of multiple stresses at the molecular level and at higher levels of biological organization [6–9]. Such an approach is crucial as we need to know how plants adapt to novel environmental factors in the context of co-occurring stresses [10].

Insect herbivory is a major biotic stress under natural conditions. Therefore, plants have evolved sophisticated constitutive and inducible defenses to resist or reduce the effects of insect attack [11]. Several studies have shown that plants subjected to abiotic stress or nutritional limitation differentially affect the performance and behavior of insects [12–17]. In addition to insects, plant pathogens are a major threat to plant growth and survival, but also impact on the colonization by and performance of herbivores feeding on pathogen-infected plants [18, 19]. As biotrophic and necrotrophic phytopathogens exhibit distinct infection pathways, they induce different plant responses [20]. Their effects on plants may influence the phytochemical environment of the insect attacker in different ways. Moreover, pathogen effects on plant resistance to insects will depend on the biology of the herbivore, e.g., whether it is a phloem feeder or a chewing larva [19]. For example, the necrotrophic pathogen *Botrytis cinerea* inhibited the development, fecundity and survival rate of the aphid *Aphis fabae* in *Vicia faba,* whereas the biotrophic fungus *Uromyces viciae-fabae* enhanced aphid performance [21]. Most interestingly, the effect of combined *B. cinerea* and *U. viciae-fabae* stress on aphid performance seemed to fluctuate depending on the order of infection [21]. In contrast, *B. cinerea* pretreatment had no significant effect on further performance of *Pieris rapae* caterpillars in Arabidopsis [22]. Tomato plants challenged by *Pseudomonas syringae* reduced *Spodoptera exigua* growth, whereas tomato mosaic virus increased caterpillar performance but decreased aphid colonization [23]. *Manduca sexta* larvae feeding on *Nicotiana attenuata* plants treated with the bacterial quorum-sensing *N*-acylhomoserine lactone were significantly heavier than on untreated plants. This effect was attributed to an inhibition of plant defenses against herbivores [24]. Oviposition by *Pieris brassicae* inhibited growth of *P. syringae* strains in Arabidopsis [25]. Furthermore, *P. brassicae* larvae showed a poor performance on *P. syringae*-infected Arabidopsis plants, suggesting that insect eggs inhibit plant defenses for the benefit of their progeny [25]. In summary, the outcome of a biotic pretreatment on herbivore performance is difficult to predict and depends primarily on the severity and duration of the infection, attack strategies of the pathogens and herbivores involved, and the plant species that is attacked.

Induced defenses are controlled by phytohormones. Biotrophic pathogens, which obtain nutrients from living tissues activate mainly the salicylic acid (SA) pathway, while necrotrophs obtaining nutrients from dead host tissues and chewing herbivores activate especially the jasmonic acid (JA) and ethylene (ET) pathways [26]. These pathways regulate the expression of defense genes that provide specific resistance to the attacker. The existence of antagonism between the SA and JA pathways is well established [26]. It is thought to modulate prioritization of defense allocation towards different attackers [27, 28] but is also the target of defense manipulation by plant pathogens and insect herbivores [26, 29–33]. Stimulation of the SA pathway attenuated plant response to generalist herbivores, i.e. herbivores feeding on plant species in different plant families, but had no effect on the specialist *P. brassicae*, which primarily feeds on plant species within the Brassicaceae family [34, 35]. At the molecular level, treatments with SA or pathogens that enhance SA levels reduced the expression of the anti-herbivore *VSP2* in Arabidopsis [36, 37]. Pathway cross talk may thus represent a crucial component of plant responses to combined stresses.

Transcriptome analyses have been conducted to better understand plant responses to multiple stresses. For instance, one study analyzed transcriptomic differences in ten ecotypes of Arabidopsis challenged by single or dual (a) biotic stress combinations. The authors concluded that the majority of changes in gene regulation in response to combined stresses were not predictable using expression profiles from single treatments [38]. Drought or flooding pretreatment significantly modified the transcriptome signature of *Solanum dulcamara* plants infested with *S. exigua* [16]. Simultaneous attack by sap-feeding and chewing herbivores in *N. attenuata* triggered a transcriptional response that was distinct from those in response to single attackers [39]. An overview of 33 different combined stresses revealed that each treatment seems to generate a unique response, reflecting the plant’s ability to specifically adapt to a changing and complex environment [8]. The same conclusion was reached for the impact of combined stresses at the metabolomic and proteomic level, with several unique metabolites and proteins accumulating after multiple stresses but not after single stresses [8, 40]. However, Arabidopsis plants challenged by both nematodes and drought responded primarily to drought [41].

Thus, plant responses to multiple stresses are interconnected and result in complicated and unpredictable outcomes. More studies on plant responses to combined stress conditions are critical to understand the effects of these interactions. This requires analysis at multiple levels, transcriptional and hormonal responses, defense compound accumulation and ecological consequences, using different plant species. Here, we investigated the effects of combined biotics stresses on plant transcriptomic changes, changes in plant hormones and metabolites and insect performance, using and ecologically relevant system. We selected the wild annual crucifer, *Brassica nigra*, subjected to feeding by a naturally associated lepidopteran pest, caterpillars of the large cabbage white *P. brassicae*, alone or in combination with a second stress. Combined stresses consisted of a pretreatment with *P. brassicae* egg extract, the cabbage aphid *Brevicoryne brassicae*, or the necrotrophic bacterial phytopathogen *Xanthomonas campestris* pv. *raphani* (*Xcr*), followed by caterpillar herbivory. All stresses used here occur naturally on *B. nigra* in the field [42, 43]. Whereas plant defenses against chewing larvae are primarily regulated by the JA pathway, eggs and aphids activate primarily the SA pathway [35, 44–46], and defense against *Xcr* is mediated by SA, JA and ET [47]. Given the known mutual antagonistic actions of these signaling pathways, we were expecting significant effects of a primary stress on the responses to *P. brassicae* larvae. Interactions of *B. nigra*-attacker interactions are well-investigated at the ecological level under field conditions where multiple attackers occur [43, 48, 49]. However, much less is known about the mechanistic aspects of the responses of *B. nigra* plants to single as compared to combined stresses. This is the topic of the present study.

## Results

### Effect of combined stresses on transcriptional responses to herbivory

We used whole-genome Arabidopsis CATMA microarrays [50, 51] to assess gene expression changes in *B. nigra*. Previous studies have shown that Arabidopsis microarrays can be successfully used to study transcriptional responses of *Brassica oleracea* or *B. nigra* [52, 53]. After 1 day of feeding by *P. brassicae* larvae on *B. nigra*, 218 genes were significantly upregulated (log_2_ > 0.585, *P* < 0.05) and 49 genes were significantly downregulated (log2 > −0.585, *P* < 0.05) (Additional file 1: Table S1). Gene ontology (GO) search of the upregulated genes revealed a highly significant enrichment of terms including response to wounding (GO:0009611), response to stress (GO:0006950), response to jasmonic acid stimulus (GO:0009753), response to biotic stimulus (GO:0009607), response to chitin (GO:0010200), defense response (GO:0006952), jasmonic acid biosynthesis (GO:0009695), oxylipin biosynthesis (GO:0031408), secondary metabolic process (GO:0019748). Downregulated genes were enriched in terms like photosynthesis (GO:0015979), cellular metabolic process (GO:0044237), nitrogen metabolism (GO:0034631, GO:0044271), chloroplast (GO:0005907, GO:0044434) (Additional file 2: Table S2). This transcriptional signature confirms results from previous studies on the response to caterpillar herbivory in other plant species, which have identified a crucial role for the jasmonate pathway in inducing anti-insect defense genes and observed a downregulation of photosynthesis-related genes [45, 54–59]. Because we used Arabidopsis microarrays, some more distantly related *B. nigra* defense genes may have been missed in the hybridization procedure. A more exhaustive list of insect-responsive genes will await transcriptome analyses by RNA sequencing once a *B. nigra* reference genome is available.

**Fig. 1:**
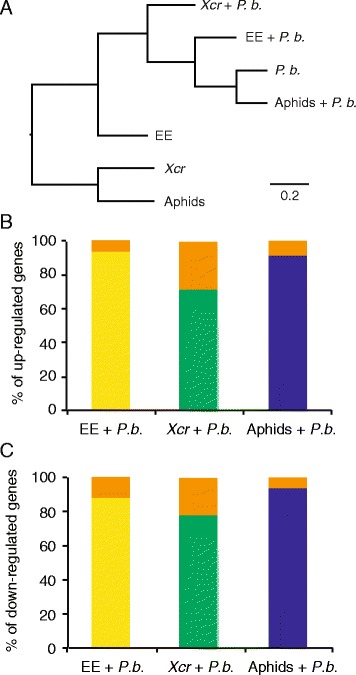
Expression profiles in response to single and combined stresses in *Brassica nigra* plants. **a** Correspondence analysis of expression profiles including all induced or repressed genes in at least one experiment (−0.585 < log_2_ ratio > 0.585, *P* < 0.05, *n* = 961). Clustering and node length calculations were performed with MultiExperiment Viewer 4.8.1 using Pearson’s correlation. **b** Proportion of *P. brassicae*-upregulated genes that are also upregulated during a combined stress. Each bar segment (*yellow*, *green*, *blue*) represents a different combined stress. The proportion of genes specifically induced by *P. brassicae* is shown in orange. The number of genes regulated by herbivory (upregulated, *n* = 218; downregulated, *n* = 49) is set to 100%. (**c**) Proportion of *P. brassicae*-downregulated genes also downregulated during a combined stress. *P.b.*, *P. brassicae* larvae; EE, *P. brassicae* egg extract; *Xcr, Xanthomonas campestris* pv. *raphani*; Aphids, *Brevicoryne brassicae*

Then, to investigate how a biotic pretreatment may affect *P. brassicae*-induced transcriptome changes, we challenged *B. nigra* plants with *P. brassicae* egg extract, the bacterial pathogen *Xcr*, or *B. brassicae* aphids before adding *P. brassicae* larvae for 24 h. As control experiments, we subjected *B. nigra* plants to each single stress. Strikingly, an expression-based clustering analysis of all experiments showed that transcriptomes from the three combined stress treatments were grouped with the transcriptome of *P. brassicae* larval treatment, whereas transcriptomes from egg extract, the bacterial pathogen, or aphid single treatments were clearly separated (Fig. 1a). Indeed, from the list of 218 uregulated and 49 downregulated genes after herbivory alone, 206 (94%), repectively 43 (88%), were still similarly regulated after egg extract pretreatment, 155 (72%), respectively 38 (78%), after pathogen pretreatment, and 201 (92%), respectively 46 (94%), after aphid pretreatment, indicating that the biotic pretreatments applied had a weak effect on the subsequent transcriptional response to herbivory (Fig. 1b, c). Analysis of the 50 top up- and downregulated genes after single treatment with caterpillars showed that expression of 48, respectively 42 genes, did not differ significantly between single or combined stress with egg extract. Similarly, 36 upregulated and 46 downregulated genes were not expressed differently between herbivory and combined stress with aphids (Additional files 3, 4: Figures S1, S2). However, in the case of pathogen pretreatment, 22 of the top-50 genes showed a significantly reduced induction, including known JA-regulated genes like *LOX3*, *CORI3*, and *OPR3*, suggesting that bacterial infection inhibits defense against herbivory (Additional file 3: Figure S1).

**Fig. 2:**
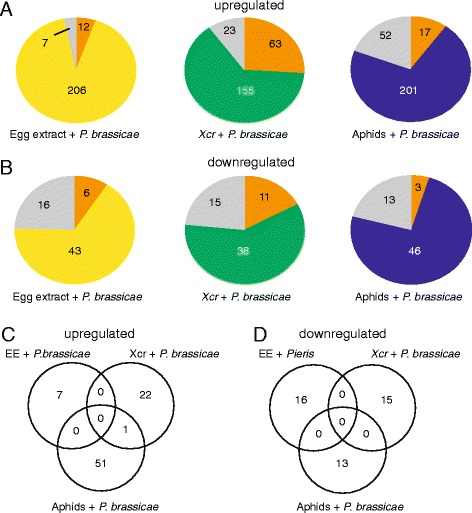
Number of **a** upregulated and **b** downregulated genes in response to single and combined stresses. Number of genes differentially regulated after combined *P. brassicae* feeding and *P. brassicae* egg extract (*yellow*), *Xanthomonas campestris* pv. *raphani* (*green*) and *Brevicoryne brassicae* (*blue*) are indicated. Number of genes specifically regulated by *P. brassicae* feeding (*orange*) and specifically regulated by a combined stress (*grey*) is also indicated. **c**, **d** Distribution of genes specifically regulated by a combined stress shows very little overlap

A combination of stresses may activate genes that are normally not regulated during single stresses. To identify a specific signature of a combined stress, we searched for genes that were significantly induced or repressed only in the three dual-stress treatments (egg extract/caterpillars, pathogen/caterpillars/, or aphids/caterpillars). There were respectively 7, 23, and 52 upregulated genes and 16, 15 and 13 downregulated genes meeting these criteria. Strikingly, a comparison of these combined-stress-specific genes indicated that only one gene was commonly regulated in egg extract/caterpillars and pathogen/caterpillars while other genes were specifically regulated by each combination of stresses (Fig. 2). A GO search of the combined-stress responsive genes did not reveal enrichment of any particular or conserved biological process (Additional files 5, 6: Figures S3, S4). These results indicate that there is no typical transcriptional signature of a combined stress but that each combination activates a relatively small number of additional genes.

### Effect of combined stresses on larval performance and plant defense compounds

**Fig. 3:**
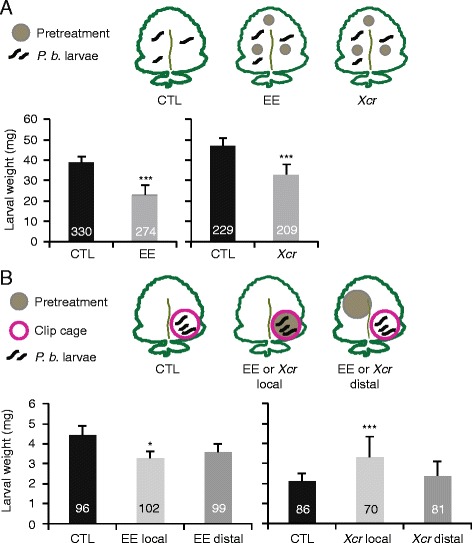
Impact of pretreatment on insect performance. **a** Larval weight of *P. brassicae* feeding on 5-week-old *B. nigra* plants pretreated for 3 days with *P. brassicae* egg extract or *Xanthomonas campestris* pv. *raphani* (*Xcr*) was measured after 7 days of feeding. Values (± SE) are the mean of independent experiments (*P. brassicae*: CTL/EE, *n* = 5; CTL/*Xcr*, *n* = 4). The total number of larvae is indicated in each column. Significant differences between control and pretreatment are indicated (linear mixed model, ***P < 0.001). **b** Larval weight of *P. brassicae* feeding on 5-week-old *B. nigra* plants pretreated for 3 days with *P. brassicae* egg extract (EE) or *Xanthomonas campestris* pv. *raphani* (*Xcr*) was measured after 4 days. Larvae placed in clip cages were feeding on the treated site (local) or adjacent to the treated site (distal). Values (± SE) are the mean of four (CTL/EE) or three (CTL/*Xcr*) independent experiments. The total number of larvae is indicated in each column. Significant differences between control and pretreatment are indicated (linear mixed model, ***P < 0.001, **P < 0.01, *P < 0.05)

Performance of *P. brassicae* caterpillars was also measured in terms of weight gain on plants pretreated with egg extract or the pathogen and on untreated plants. When caterpillars were feeding freely on entire leaves pre-treated with egg extract or the pathogen, their weight gain was significantly reduced compared to that on control plants (linear mixed model (LMM), *P*_[egg extract]_ = 2*10^−16^ and pathogen, *P*_[pathogen]_ = 2*10^−8^) (Fig. 3a). To test whether altered insect performance on pretreated plants correlated with changes in defense signals and metabolites, we quantified SA, JA, and glucosinolate (GS) concentrations. GS are potent defense compounds in brassicaceous plants, effective against generalist insects, that accumulate in response to herbivory [60–64]. However, results from whole-leaf analyses showed that concentrations of JA and total GS were not significantly different in leaves that were pretreated with egg extract or the pathogen followed by caterpillar feeding and in leaves exposed to caterpillar feeding alone (Two-way ANOVA, *P*_[JA, egg extract]_ = 0.62, *P*_[JA, pathogen]_ = 0.17, *P*_[GS, egg extract]_ = 0.46, *P*_[GS, pathogen]_ = 0.41) (Additional file 7: Figure S5). For SA, the presence or absence of caterpillar feeding did not alter the significant accumulation in response to egg extract or pathogen treatment (Two-way ANOVA, *P*_[SA, egg extract]_ = 0.20, *P*_[SA, pathogen]_ = 0.49) (Additional file 7: Figure S5).

Since the difference in larval performance between untreated and pretreated plants was not easily explained by transcriptomic data, by changes in defense hormonal signaling, or by GS accumulation in whole *B. nigra* leaves, we decided to study the effect of pretreatment relative to the feeding site of the caterpillars. The rationale was that locally induced changes within the leaf may account for the observed effects. Noteworthy, a recent study on maize has reported statistically significant higher concentration of defense metabolites, i.e. 1,4-benzoxazin-3-ones, in young leaves compared to old ones and that this was negatively correlated with insect performance [65]. We modified the experimental design by constraining larvae in clip cages locally or distally, relative to the pretreatment site. Caterpillar weight gain was significantly reduced after egg extract pretreatment, but only when caterpillars were forced to feed on the pretreatment site (LMM, *P*_[local]_ = 0.029; *P*_[distal]_ = 0.37) (Fig. 3b). The effect was similar to the whole-leaf response (Fig. 3a). In contrast, caterpillars performed significantly better on pathogen-pretreated site than on control leaves (LMM, *P* = 9*10^−7^) but their weight was not different when forced to feed distally from the pretreatment site (LMM, *P* = 0.22), suggesting for instance a local suppression of defenses by bacterial effectors (Fig. 3b). This result was different from the result of the whole-leaf experiment, where caterpillar performance was reduced on pathogen-pretreated leaves (Fig. 3a). Thus, the respective localization of pathogen pretreatment and caterpillar feeding site clearly impacted the effect of the pathogen on insect performance.

**Fig. 4:**
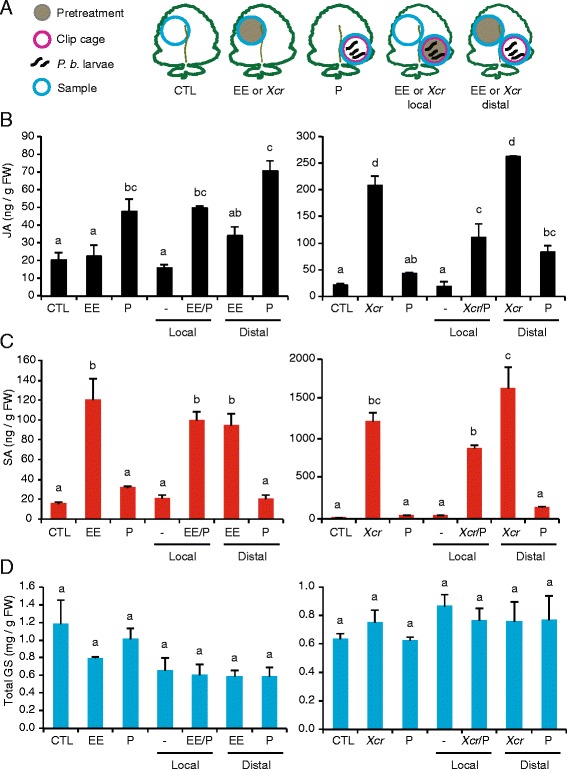
Quantification of defense signals and glucosinolates. **a** Experimental design. Quantification of jasmonic acid (JA) **b**, salicylic acid (SA) **c** and total glucosinolates (GS) **d** in single and combined stress. Leaf discs (blue circle) were collected on 5-week-old *B. nigra* plants pretreated for 3 days with *P. brassicae* egg extract (EE) or *Xanthomonas campestris* pv. *raphani* (*Xcr*) and further challenged with *P. brassicae* (P) larvae feeding for 24 h at the site or distal to the site of treatment. Controls consisted of untreated plants (CTL) or plants exposed to a single treatment. -, sample distal from the combined treatment. Values (± SE) are the mean of three independent experiments. Letters indicate significant difference between treatments (ANOVA followed by Tukey’s honest significant difference test, *P* < 0.05)

To further correlate insect performance and site of treatment with defense compound and signaling hormone accumulation, we quantified SA, JA, and GS concentrations in leaf tissues collected from untreated plants, and from pretreated plants at the site where the pretreatment stress was applied. In plants that were also exposed to caterpillar feeding, leaf tissues were collected from the areas where the caterpillars were constrained, so at the site of pretreatment or at a site distal from the pretreatment application (Fig. 4a). In the experiment with egg extract, JA accumulated only in response to herbivory. Egg-extract pretreatment itself did not cause JA accumulation and did not alter caterpillar-induced JA concentration (ANOVA, *F* = 13,31, *P* = 0.0001) (Fig. 4b). In contrast, pretreatment with the pathogen resulted in a 10-fold increase of JA, whereas JA concentrations in leaf tissues exposed to caterpillar feeding alone were not significantly different from those found in control plants (ANOVA, *F* = 68.85, *P* = 5*10^−7^) (Fig. 4b). JA was induced equally (5-fold) when caterpillars were feeding distally from the site where the pathogen was applied, and when caterpillars were feeding at the same site (Fig. 4b). Egg extract and pathogen pretreatments significantly induced SA, only at the treatment site. Moreover, there was no change in SA concentrations in response to herbivory, and caterpillars did not affect egg extract- or pathogen-induced SA concentrations (ANOVA, *F*_[egg extract]_ = 18.37, *P*_[egg extract]_ = 2*10^−5^, *F*_[pathogen]_ = 66.18, *P*_[pathogen]_ = 5*10^−8^) (Fig. 4c). Thus, *B. nigra* leaves respond locally to biotic challenges by accumulating distinct JA or SA concentrations depending on the biotic stress. Furthermore, the SA response to combined stresses did not differ from the hormonal response to single stresses, whereas it did for JA.

As with whole-leaf experiments, total GS concentrations did not change significantly between control and treated plants (ANOVA, *F*_[egg extract]_ = 2.71, *P*_[egg extract]_ = 0.058, *F*_[pathogen]_ = 0.66, *P*_[pathogen]_ = 0.68) (Fig. 4d). Among the 13 glucosinolates that were identified and quantified (Additional file 8: Table S3), sinigrin contributed 91% to 96% of the total GS content in different treatments. Consistent with a lack of GS accumulation after biotic stress in *B. nigra*, we observed that expression of 22 out of 27 GS biosynthesis genes was not significantly enhanced in response to single or combined stresses (Additional files 1, 9: Table S1, Figure S6). This contrasts with the coordinated induction of genes involved in all steps of GS biosynthesis in Arabidopsis after herbivory [64].

### Effect of combined stresses on SA/JA cross talk

**Fig. 5:**
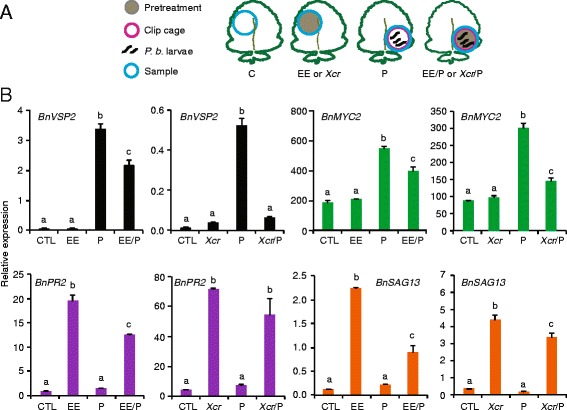
Expression of JA- and SA-related genes. **a** Experimental design. **b** Expression of *B. nigra* genes was measured by QPCR and normalized to the housekeeping gene *BnSAND*. Leaf discs (blue circle) were collected on 5-week-old *B. nigra* plants pretreated for 3 days with *P. brassicae* egg extract (EE) or *Xanthomonas campestris* pv*. raphani* (*Xcr*) and further challenged with *P. brassicae* (P) feeding for 24 h at the site of treatment. Untreated plants (CTL) or plants exposed to a single stress were included. Means (± SE) of three technical replicates are shown. These experiments were repeated at least twice with similar results. Different letters indicate significant differences (two-way ANOVA followed by Tukey’s honest significant difference test, *P* < 0.05)

Since exposure to egg extract, pathogen treatment and caterpillar feeding triggered SA and JA accumulation to different extents, we decided to investigate the known SA/JA antagonism in response to combined stresses. We designed QPCR primers for *B. nigra* sequences related to *VSP2* and *MYC2*, which are JA- and herbivory-regulated genes [56], and for *PR2* and *SAG13*, which are SA- and egg-regulated genes [66]. In single stress treatments, *BnVSP2* and *BnMYC2* expression were significantly upregulated in tissues exposed to caterpillar feeding, but not in tissues treated with egg extract or the pathogen (Two-way ANOVA, *P*_[VSP2, egg extract]_ = 0.01, *P*_[VSP2, pathogen]_ < 0.0001, *P*_[MYC2, egg extract]_ = 0.04, *P*_[MYC2, pathogen]_ < 0.0001) (Fig. 5). Contrastingly, *BnPR2* and *BnSAG13* were significantly upregulated in tissues treated with egg extract or the pathogen, but not in tissues exposed to caterpillar feeding (Two-way ANOVA, *P*_[PR2, egg extract]_ < 0.0001, *P*_[PR2, pathogen]_ < 0.0001, *P*_[SAG13, egg extract]_ < 0.0001, *P*_[SAG13, pathogen]_ < 0.0001) (Fig. 5). Interestingly, for combined stresses we found that both egg extract and pathogen pretreatments led to a significantly reduced induction of insect-responsive *BnVSP2* and *BnMYC2*. Combined stresses also reduced the induction of egg extract- or pathogen-responsive *BnSAG13*, whereas *BnPR2* induction was only inhibited by egg extract pretreatment (Fig. 5). These results suggest that under dual-stress conditions a combined accumulation of SA and JA in response to either egg extract or pathogen pretreatment followed by herbivory negatively affects specific JA-and SA-responsive genes. We thus observed a consistent and reciprocal SA/JA cross talk in *B. nigra*, in response to treatment with egg extract or the pathogen followed by caterpillar feeding.

## Discussion

Exposure to two or more biotic stresses can either be more detrimental than a single stress or, conversely, have an attenuating effect. The ability of plants to recognize and respond to combined and specific stresses appears thus to be important, especially if stresses, such as pathogens and herbivores, trigger different plant defense pathways. Few studies have been conducted on whole-genome responses under multiple stress conditions, and gene expression studies often focused on the plant model Arabidopsis. Here, *B. nigra,* which is also a brassicaceous plant species, was used to investigate how plants respond to combined stresses. Surprisingly, transcriptomic responses of *B. nigra* to different pretreatments followed by *P. brassicae* herbivory revealed that the first stress has only a weak impact on transcriptional responses to the second stress. In addition, no genes common to a combined stress could be identified. It was recently found that the effect of a previous exposure to *B. cinerea* or to drought only slightly changed Arabidopsis transcriptional response to *P. rapae* feeding, suggesting that plants prioritize a response to the second stress [22]. Similarly, Arabidopsis transcriptome after *P. brassicae* feeding was not affected by pre-exposure to *P. brassicae* eggs, although it was impacted by a cold pretreatment [67]. However, a detailed time-course analysis revealed that pre-exposure shifts the timing of caterpillar-induced responses. Plants responded faster to *P. rapae* if they were preceded by a drought or *B. cinerea* treatment [59], indicating that timing of the response needs to be considered. Atkinson and coworkers [41] postulated that during multiple attack, plants respond preferentially to the most damaging stress (see also [68]). In Arabidopsis challenged by drought and/or nematodes, 96% of differentially regulated genes were shared between the combined stress and water stress, whereas only 2% overlapped with nematode feeding [41]. We hypothesize that *B. nigra* prioritized a response to caterpillar feeding rather than to aphids, eggs or bacteria for the benefit of its own fitness, as *P. brassicae* caterpillars are known to be voracious feeders on brassicaceous plants; these caterpillars are florivorous when reaching the second and subsequent instars, thus reducing fitness directly [69]. Also at the level of the metabolome, changes in *B. nigra* plants exposed to *B. brassicae* aphids and/or *P. brassicae* caterpillars were the strongest in response to feeding by caterpillars both when feeding alone or together with the aphids on the same leaf [68]. It would be interesting to perform reciprocal experiments to see if the transcriptome of *P. brassicae*-pretreated plants is dominated by the signature of a second biotic stress or if plants prioritize the response to herbivory over other stresses. A recent transcriptome study on Arabidopsis plants infected by *Botrytis cinerea* with or without prior herbivory suggests that the first hypothesis is more likely [59]. Another testable hypothesis is that plants respond to the most severe stress, irrespective of the order of attack.

We observed that pretreatment with the biotrophic pathogen *Xcr* had a measurable effect on the *P. brassicae* transcriptome. Indeed, ca. 30% of insect-induced genes, including JA-regulated genes, were significantly less induced in response to the combined stress, but at the same time weight-gain of *P. brassicae* caterpillars was reduced on pathogen-infected plants when feeding on the entire leaf or when restricted to feed distally from the site where the pathogen-pretreatment was applied. Since *Xcr* single treatment triggered SA accumulation, we hypothesize that SA/JA cross talk was responsible for this attenuation of gene expression. Our targeted analysis of the JA markers *BnVSP2* and *BnMYC2* confirmed this observation, but the insect performance assay also indicated that attenuation of these genes does not have negative consequences for plant defense against *P. brassicae*. Caterpillar weight gain was also reduced on plants pretreated with an egg extract. SA-responsive genes, including *PR1*, were clearly up-regulated by egg extract treatment indicating that the SA pathway was activated by eggs, like in Arabidopsis [35, 66]. This apparent absence of SA/JA cross talk at the whole-genome level may be explained by a relatively less strong response to *P. brassicae* eggs. Indeed, we found a much higher SA accumulation after *Xcr* pathogen than after *P. brassicae* egg extract treatment. In addition, whole-leaf analysis may have diluted a localized response since we detected a localized suppression of *BnVSP2* and *BnMYC2* expression after egg extract treatment. Cross talk between defense signaling pathways is known to strongly modulate the outcome of combined biotic stresses [26]. Here, we also showed that pathogen and egg extract pretreatments inhibited both induction of JA- and SA-regulated genes in response to additional feeding by *P. brassicae* caterpillars, suggesting that SA/JA cross talk reduced the transcription of some genes considered important in plant defense against insects.

Although single *Xcr* infection led to a strong SA and JA accumulation, it is noteworthy that only SA-regulated *BnPR2* and *BnSAG13* were induced but not JA-regulated *BnVSP2* and *BnMYC2*. Moreover, SA/JA cross talk effects were most pronounced on *BnVSP2* and *BnMYC2* transcript levels in response to combined *Xcr*/herbivory stresses, indicating that SA strongly influenced the JA pathway in this particular context. Depending on the hormonal context, accumulation of a defense signal is thus not necessarily correlated with the induction of downstream genes. Conversely, pathogen- and egg-extract induction of SA-signaling related genes *BnPR2* and *BnSAG13* was inhibited by caterpillar feeding, suggesting that the reciprocal JA/SA cross talk was also operating. The consequence of such cross talk on susceptibility to *Xcr* infection was not tested but would be an interesting topic for future research. Thus, JA and SA activation and their mutualistic antagonistic effects may depend on the strength or the nature of the treatment. For example, a study in Arabidopsis reported a synergistic or antagonistic effect on JA- and SA-induced genes if plants were treated with low or high concentrations of each hormone, respectively [70]. In addition, plant responses to herbivory are known to be dynamic and may depend on the sampling time [59, 71]. It will thus be interesting in future experiments to see if our observations on SA/JA cross talk at a single time point robustly underlie the outcome of the combined interactions. In conclusion, although the emerging picture is that of a domination of the most recent stress on the transcriptional response [22, 59], it would be interesting to confirm this hypothesis by extending the range of reciprocal combinations of biotic and abiotic stresses, including time-course analyses.

We found that insect performance differed between treatments, suggesting that plant resistance status after combined stresses is difficult to predict based on transcriptome, defense hormone profiles or defense pathway cross talk. Indeed, we observed that insect performance after *P. brassicae* egg-extract application was decreased. In Arabidopsis, we previously showed that *P. brassicae* egg deposition had no effect on performance of the specialist *P. brassicae* [35]. Other studies with Arabidopsis and *B. nigra* revealed that *P. brassicae* performed less well or equally in the presence of eggs, depending on the species identity of the egg donor [67, 72–74]. Similar results were found for *P. brassicae* feeding on other wild brassicaceous species, i.e. *Brassica oleracea*, *Moricandia moricandioides* and *Sinapsis arvensis* exposed to *P. brassicae* eggs [43]. On the contrary, the generalist *S. littoralis* performed better on plants already treated with *P. brassicae* egg extract or after natural oviposition [35, 75] but no effect was found for eggs of the generalist *Mamestra brassicae* on subsequent *M. brassicae* larval performance [72]. Thus, whether insect eggs induce plant defenses is context-specific. Similarly, whether there is an effect of exposure to a biotrophic pathogen is context specific [25, 76] and may also depend on the virulence level of the pathogen [77].

Furthermore, *P. brassicae* caterpillars feeding freely on *Xcr*-pretreated leaves gained less weight than caterpillars feeding on an untreated leaf. Thus, at the whole-leaf level, *Xcr* pretreatment impacted plant defense responses similarly to the egg extract pretreatment, although the underlying mechanism might have been different. A study on *Capsicum annuum* L. reported an enhanced performance of *S. exigua* larvae on plants infected with *X. campestris* pv. *vesicatoria* [76]. In contrast, performance of *P. brassicae* larvae was reduced on Arabidopsis infected with *Pseudomonas syringae* pv. tomato [25]. Again, insect performance on plants infected with phytopathogens seems to be variable.

Surprisingly, compared to whole-leaf bioassays, insect performance assays yielded somewhat contrasting conclusions using clip cages to restrict feeding by caterpillars on specific sites on a leaf. Whereas egg-extract treatment impacted larvae similarly regardless whether they were feeding freely on the whole leaf or locally in a clip cage, *P. brassicae* caterpillars feeding on *Xcr*-infiltrated leaf area were larger than those constrained to feed on a non-infected zone or on an untreated leaf. This observation could be explained by our finding of a local inhibition of JA-dependent defense gene expression after *Xcr* pretreatment, although a restricted feeding on egg-extract pretreated tissues did not result in enhanced insect performance. Hence, other factors likely contribute to a localized effect. *Xcr* infection triggered a local accumulation of both SA and JA while *P. brassicae* egg-extract treatment only triggered SA accumulation. Insect herbivores tend to avoid defended leaf areas [78], which they could not do under the constrained clip-cage conditions. Indeed, when given the choice we noticed that *P. brassicae* larvae avoided egg-treated and *Xcr*-infected zones (Additional file 10: Figure S7). This is intriguing with regard to the opposite performance of larvae when feeding on egg-treated or *Xcr*-infected zones. This finding of larval selective feeding deserves further investigation.

Differential activation of the SA and JA signaling pathways may affect metabolite composition. However, we did not observe a differential GS accumulation between treatments. A similar finding was observed after combined ozone and *P. brassicae* treatment in *B. nigra*, although larvae grew less well on ozone-pretreated plants [79]. Contrasting results were reported in the study by Ponzio et al. [68] where the total GS concentration significantly increased in response to feeding by *P. brassicae* caterpillars. Furthermore, induction of GS under dual stress conditions with caterpillars and *B. brassicae* aphids depended on the density of the aphids. The difference in caterpillar densities per leaf, i.e. Thirty in the Ponzio et al. study [68] and 10 here, may explain this discrepancy. As postulated previously [80], other defense compounds may play a crucial role in influencing *P. brassicae* performance on Brassicaceous plant species*.* The recent identification of flavonoid compounds that negatively impact *P. brassicae* caterpillar performance in Arabidopsis supports this conclusion [81, 82]. In addition, since ET is an important regulator associated with *Xcr* in Arabidopsis [47], local ET signaling may be involved in the local effect of *Xcr* on *P. brassicae* performance. Moreover, plant nutritional quality at the treatment site could also play a role. It may negatively correlate with insect performance on egg-treated sites but positively on *Xcr*-treated sites. For instance, leaf carbohydrate content was found to be controlled by JA and mediated plant susceptibility to an adapted herbivore in *Nicotiana attenuata* [83].

## Conclusions

Our transcriptome analysis of *B. nigra* in response to combined stress treatments revealed that the second stress dominates the transcript signature, although pretreatments clearly impacted how plants resisted an herbivore attack. Measurement of defense-signaling hormones and transcript levels of defense marker genes in response to multiple attack by different stresses do not necessarily predict the plant’s defense response in a straightforward fashion. Future studies should include more marker genes representing different steps along the molecular sequence of events. Our results show that under conditions of multiple stress the plant responds highly specifically to each stress combination. Contrasting responses strongly suggest that we need to better integrate responses at different levels of biological organization, to consider local versus distant plant responses within a leaf, and to measure the accumulation of a range of (defense) metabolites determining nutritional quality when trying to correlate plant traits with insect performance.

## Methods

### Biological material

Seeds of *Brassica nigra* were collected from a wild population in Wageningen (The Netherlands) [43]. Plants were grown in soil in growth chambers (16 h light, at 25 °C day, 22 °C night, 60% relative humidity) under white fluorescent light (170 μmol m^−2^ s^−1^). Seeds were stratified for 3 days at 4 °C after sowing. The soil contained 65% humus, 10% sand, 15% perlite and 10% silt. Growth conditions were the same in the different bioassays described below.

*Xanthomonas campestris* pv. *raphani* (*Xcr*) (formerly classified as *X. campestris* pv. *armoraciae*) was obtained from the Plant-Microbe Interactions group of Utrecht University (The Netherlands) and was originally acquired from the Department of Plant Pathology at Ohio State University (USA). The pathovar identity was confirmed by pathogenicity assays and PCR. Bacteria were grown in 10 ml of liquid King B culture medium (20 g / l peptone (Sigma-Aldrich), 1.5 g / l dipotassium hydrogen phosphate, 1.5 g / l magnesium sulfate heptahydrate, 12 g /l agar, at a final pH of 7.2) supplemented with rifampicin (25 μg/ ml) and grown in a shaker at 28 °C, 200 rpm, during 48 h. *Xcr* culture was centrifuged at 7000 rpm during 2 min. The supernatant was discarded and the pellet was washed and re-suspended in 10 mM MgCl_2_ and centrifuged again at 7000 rpm during 2 min. The supernatant was discarded and the pellet diluted in 10 mM MgCl_2_ and adjusted to an OD 600 of 0.07 to obtain a concentration of 10^7^ cfu/ml.

*Pieris brassicae* was reared on Brussels sprout plants (*Brassica oleracea var. gemmifera*) in 1 m^3^ cages in a greenhouse (25 ± 5 °C, 60 ± 5% RH, 16/8 h light-dark cycle) at Lausanne University (Switzerland). Eggs were removed manually from the plants and crushed with a pestle in Eppendorf tubes. After centrifugation (15′000 *g*, 3 min), the supernatant (egg extract) was stored at −20 °C.

*Brevicoryne brassicae* aphids were reared on *B. oleracea* var. *gemmifera* in a greenhouse (22 ± 3 °C, 65 ± 5% RH, 16/8 h light-dark cycle) at Wageningen University (The Netherlands), where all experiments with aphids were also performed. *B. nigra* plants were grown in peat soil (Lentse potgrond no. 4, Lent, The Netherlands).

The pest species *P. brassicae* and *B. brassicae*, and *B. nigra* plants were collected in the wild in The Netherlands. This complies with national legislation as The Netherlands allows free access to its biodiversity under the Nagoya Protocol. Correct identification of *B. nigra* was confirmed by Dr. E. H. Poelman (Department of Plant Sciences, Wageningen University, The Netherlands). Seeds of *B. oleracea* var. *gemmifera* were obtained commercially from Semences Zollinger (1897 Les Evouettes, Switzerland) or Syngenta Seeds (2678 LV De Lier, The Netherlands).

### Plant treatments

The overall experimental design is summarized in Table S4 (Additional file 11: Table S4). Plants were 5 weeks old when exposed to the various treatments. Pretreatments with egg extract and the pathogen were applied to the three youngest fully developed leaves of three plants. Aphids were applied to a single leaf, i.e. the first fully developed leaf (nine plants in total), according to a design that has been used in previous experiments with the same study system [84, 85]. For *P. brassicae* egg-extract treatment, 12 × 2 μl of egg extract were added to each of the three leaves and incubated for 72 h. This treatment was equivalent to treatments previously applied to Arabidopsis and corresponds to approximately 10–12 egg batches per leaf, each batch consisting of 20–30 eggs [35, 66]. Leaves of untreated plants were used as controls.

For infection with the bacterial pathogen, *X. campestris* pv. *raphani,* each of the three treatment leaves was subjected to three infiltrations of 10^7^ cfu/ml using a 1 ml needleless syringe and incubated for 72 h. Each infiltration zone represented a circle of 1.5 cm^2^. In control plants, the same number of 10 mM MgCl_2_ infiltrations was performed.

For treatment with *B. brassicae* aphids, 100 nymphs were placed on the youngest fully developed leaf on each of nine plants, which were incubated for 48 h. Aphids were not constrained but remained on the leaf on which they had been introduced.

Treatment with caterpillars consisted of the introduction of 10 neonate caterpillars on the three leaves that had received a pretreatment (combined stresses) or on three leaves similar in development of clean plants. Thirty neonate caterpillars were introduced on the single aphid-treated leaf or a single leaf of clean plant. Caterpillars were allowed to feed for 24 h.

All experiments were repeated independently five or more times at intervals of several weeks.

### Insect performance assays on plants pretreated with egg extract or pathogen

Five-week-old *B. nigra* plants were placed in 60 × 60 × 60 cm plastic tents (Bugdorm company) in a growth chamber (20 ± 1 °C, 65 ± 10% relative humidity, 10/14 h light-dark cycle, 100 μmol m^−2^ s^−1^). For insect bioassays performed on entire leaves, ten neonate caterpillars were placed on each of the three pretreated leaves or on three leaves of clean plants with a total of 30 caterpillars per plant. Caterpillar weight was measured after 7 days of feeding. For bioassays investigating local vs. distal effects of pretreatment, five neonate caterpillars were placed in a clip cage (36.5 × 25.4 × 9.5 mm, BioQuip Products, USA) on each of three pretreated leaves with a total of 15 larvae per plant either at the same site or a site distal from where the pretreatment was applied. Plants were pretreated as described above with egg extract or the pathogen and incubated for 3 days (see Fig. 4a for experimental design). Caterpillar weight was measured after 4 days. For all experiments, insect recovery was similar between treatments.

Each treatment was done on three different plants for each biological replicate. All experiments were repeated independently three or more times at intervals of several weeks.

### Hormone and glucosinolate analysis

Leaf tissues that were sampled for hormone (SA and JA) and GS analysis were exposed to egg extract, the pathogen, and/or caterpillar feeding as described above. Entire leaves (experiments with no constraint on caterpillar feeding) or 2.4 cm leaf discs (experiments with constrained caterpillar feeding) were harvested and frozen in liquid nitrogen. Extraction, UHPLC-QTOFMS measurement and data analysis were conducted as described earlier [86, 87]. Three independent biological replicates were analyzed for each treatment.

### Transcriptome analyses

Following treatment, entire leaves were harvested, flash-frozen in liquid nitrogen and stored at −80 °C. RNA extraction, probe labeling, hybridization onto Arabidopsis CATMAv4 microarrays, and data analyses have been published previously [51, 56, 88]. For data analysis, we used an expression threshold of log_2_ > 0.585 and < −0.585, and an unadjusted *P*-value of 0.05. FDR values are shown in supplementary data for further evaluation. GO enrichment analysis was performed with AgriGO singular enrichment analysis using hypergeometric test [89].

### Quantitative PCR

Relative gene expression was measured according to previously published procedures [35, 90]. Briefly, 500 nanograms of total RNA were transcribed to cDNA using M-MLV reverse transcriptase (Invitrogen) and oligo dT primers according to commercial instructions. cDNA synthesis was done in triplicates. QPCR analysis was performed in a final volume of 25 μl according to the Brilliant III Fast SYBR Green instruction manual (Agilent). *B. nigra* primers (Additional file 12: Table S5) were designed on conserved sequences identified by multiple alignments of genes from different species of the *Brassica* family. Sequences were obtained from the *Brassica* database (http://brassica.nbi.ac.uk/BrassicaDB/). Each primer has a Tm of 60 °C and gives an amplicons length between 100 and 250 bp in the conserved part of the cDNA strand. Primer efficiencies were evaluated by five-step dilution regression. Each amplicon produced a single band and was confirmed by Sanger sequencing. For normalization, the *BnSAND* gene was used as housekeeping gene. Similar to Arabidopsis *SAND* gene [91], its expression was stable across experiments.

## Additional files

Additional file 1: Table S1. Gene expression ratios (log_2_) for all biological replicates. (XLSX 16429 kb)

Additional file 2: Table S2. GO analysis of *P. brassicae*-regulated genes. (PDF 49 kb)

Additional file 3: Figure S1. Expression of the top-50 upregulated genes in response to *P. brassicae* feeding and combined stresses. The highest significantly upregulated genes (log_2_ > 0.585, *P* < 0.05) were extracted from microarray data (orange bars) and plotted with values from combined stresses. (A) Egg extract/*P. brassicae* larvae (yellow bars), (B) *Xanthomonas campestris* pv. *raphani*/*P. brassicae* larvae (green bars), and (C) *Brevicoryne brassicae*/*P. brassicae* larvae (blue bars). Significant differences between single and combined stress are indicated (Student’s *t*-test, ***P < 0.001, **P < 0.01, *P < 0.05). (PDF 1871 kb)

Additional file 4: Figure S2. Expression of the top-50 downregulated genes in response to *P. brassicae* feeding and combined stresses. The highest significantly downregulated genes (log_2_ < −0.585, *P* < 0.05) were extracted from microarray data (orange bars) and plotted with values from combined stresses. (A) Egg extract/*P. brassicae* larvae (yellow bars), (B) *Xanthomonas campestris* pv. *raphani*/*P. brassicae* larvae (green bars), and (C) *Brevicoryne brassicae*/*P. brassicae* larvae (blue bars). Significant differences between single and combined stress are indicated (Student’s *t*-test, ***P < 0.001, **P < 0.01, *P < 0.05). (PDF 1875 kb)

Additional file 5: Figure S3. GO analysis of genes specifically upregulated by combined stress. GO terms significantly enriched with each combined stress are shown separately. Length of the bars shows the percentage of regulated genes in the respective GO categories. (PDF 951 kb)

Additional file 6: Figure S4. GO analysis of genes specifically downregulated by combined stress. GO analysis of genes specifically downregulated by combined stress. GO terms significantly enriched with each combined stress are shown separately. Length of the bars shows the percentage of regulated genes in the respective GO categories. (PDF 918 kb)

Additional file 7: Figure S5. Quantification of defense signals and glucosinolates. (A) Experimental design. (B) Quantification of jasmonic acid (JA), salicylic acid (SA) and total glucosinolates (GS) in single and combined stress in whole 5-week-old *B. nigra* leaves. Plants were pretreated for 3 days with *P. brassicae* egg extract (EE) or *Xanthomonas campestris* pv. *raphani* (*Xcr*) and further challenged with *P. brassicae* (P) larvae for 24 h. Controls (CTL) consisted of untreated plants or plants exposed to a single treatment. Values (± SE) are the mean of three independent experiments. Letters indicate significant difference between treatments (two-way ANOVA followed by Tukey’s honest significant difference test, *P* < 0.05). (PDF 1050 kb)

Additional file 8: Table S3. Glucosinolate content in *B. nigra* leaves. (PDF 37 kb)

Additional file 9: Figure S6. Expression of glucosinolate biosynthesis genes in response to *P. brassicae* feeding and combined stresses. Values were extracted from microarray data. *P. brassicae* larvae (orange bars), egg extract/*P. brassicae* larvae (yellow bars), *Xanthomonas campestris* pv. *raphani*/*P. brassicae* (green bars), *Brevicoryne brassicae*/*P. brassicae* (blue bars). Significant differences between single and combined stress are indicated (Student’s *t*-test, ***P < 0.001, **P < 0.01, *P < 0.05). (PDF 982 kb)

Additional file 10: Figure S7. Feeding behavior of *P. brassicae* larvae in response to combined stresses. Neonate larvae were allowed to feed freely for 2 days (*P. brassicae*) on 5-week-old *B. nigra* plants pretreated for 3 days with egg extract (A) or *Xanthomonas campestris* pv. *raphani* (B). Representative images from three biological replicates are shown. Scale bar = 1 cm. (PDF 9284 kb)

Additional file 11: Table S4. Overall experimental design. (PDF 48 kb)

Additional file 12: Table S5. List of primers used for QPCR. (PDF 51 kb)
