# Supplementary material for: Combined biotic stresses trigger similar transcriptomic responses but contrasting resistance against a chewing herbivore in Brassica nigra
Source: BMC Plant Biol. 2017 Jul 17;17:127. doi: 10.1186/s12870-017-1074-7 (PMC5513356; doi:10.1186/s12870-017-1074-7)
Supplement: Supplementary file 2 — GO analysis of P. brassicae-regulated genes. (PDF 49 kb) [file 12870_2017_1074_MOESM2_ESM.pdf]

**Table S2.** GO analysis of *Pieris brassicae*-regulated genes

**upregulated genes**

| GO_acc | term_type | Term | queryitem | querytotal | bgitem | bgtotal | pvalue | FDR | genes (n≤10) |
|---|---|---|---|---|---|---|---|---|---|
| GO:0009611 | P | response to wounding | 19 | 209 | 197 | 37767 | 1.60E-17 | 1.30E-14 | At3g45140, At1g80840, At5g20230, At1g32640, At5g24770, At2g06050, At3g45640, At4g23600, At1g32920, At1g55020 |
| GO:0009605 | P | response to external stimulus | 23 | 209 | 429 | 37767 | 1.00E-15 | 4.00E-13 | At1g80840, At1g32640, At5g59820, At2g34810, At2g30020, At3g45640, At1g17420, At5g57560, At5g48850, At2g06050 |
| GO:0006950 | P | response to stress | 46 | 209 | 2320 | 37767 | 4.60E-14 | 1.20E-11 | At2g38750, At5g06870, At1g80840, At5g06320, At5g59820, At3g45640, At2g33380, At5g28540, At5g17000, At3g51920 |
| GO:0042221 | P | response to chemical stimulus | 42 | 209 | 2085 | 37767 | 4.40E-13 | 8.40E-11 | At2g38750, At3g51920, At1g80840, At1g32640, At5g59820, At3g45640, At2g33380, At5g28540, At5g17000, At2g34810 |
| GO:0050896 | P | response to stimulus | 58 | 209 | 4057 | 37767 | 9.60E-12 | 1.50E-09 | At2g38750, At5g06870, At1g80840, At5g06320, At5g59820, At3g45640, At2g33380, At5g28540, At5g17000, At3g51920 |
| GO:0009753 | P | response to jasmonic acid stimulus | 14 | 209 | 215 | 37767 | 4.50E-11 | 5.70E-09 | At3g45140, At4g23100, At5g24780, At1g32640, At5g67300, At4g23600, At1g55020, At5g24770, At1g74100, At2g34810 |
| GO:0009620 | P | response to fungus | 12 | 209 | 158 | 37767 | 2.10E-10 | 2.40E-08 | At4g23100, At1g80840, At4g31800, At2g25000, At2g33380, At3g45140, At2g44490, At2g30020, At3g25780, At1g17420 |
| GO:0010033 | P | response to organic substance | 29 | 209 | 1342 | 37767 | 6.10E-10 | 5.90E-08 | At2g38750, At3g51920, At1g80840, At1g32640, At5g59820, At3g45640, At2g33380, At5g28540, At2g34810, At1g08930 |
| GO:0009607 | P | response to biotic stimulus | 20 | 209 | 638 | 37767 | 8.60E-10 | 7.40E-08 | At3g45140, At4g23100, At1g80840, At4g31800, At5g06320, At5g24770, At2g41430, At2g25000, At2g33380, At5g28540 |
| GO:0051707 | P | response to other organism | 19 | 209 | 599 | 37767 | 1.90E-09 | 1.50E-07 | At3g45140, At4g23100, At1g80840, At4g31800, At5g06320, At5g24770, At2g41430, At2g25000, At2g33380, At1g55020 |
| GO:0010035 | P | response to inorganic substance | 12 | 209 | 279 | 37767 | 9.00E-08 | 6.30E-06 | At4g23100, At4g14880, At1g07890, At2g41380, At5g67300, At3g10920, At5g24770, At1g07600, At5g37770, At4g02380 |
| GO:0051704 | P | multi-organism process | 19 | 209 | 776 | 37767 | 1.10E-07 | 6.80E-06 | At3g45140, At4g23100, At1g80840, At4g31800, At5g06320, At5g24770, At2g41430, At2g25000, At2g33380, At1g55020 |
| GO:0031407 | P | oxylipin metabolic process | 6 | 209 | 39 | 37767 | 1.80E-07 | 1.00E-05 | At3g45140, At2g06050, At2g33380, At1g55020, At3g25780, At1g17420 |
| GO:0010200 | P | response to chitin | 9 | 209 | 151 | 37767 | 2.90E-07 | 1.60E-05 | At1g80840, At4g31800, At4g17230, At1g32640, At5g67300, At5g59820, At5g64660, At3g45640, At1g08930 |
| GO:0006952 | P | defense response | 18 | 209 | 766 | 37767 | 4.10E-07 | 2.10E-05 | At5g06870, At1g80840, At5g24780, At5g06320, At5g24770, At2g25000, At1g52040, At4g23100, At1g55020, At1g74100 |
| GO:0044272 | P | sulfur compound biosynthetic process | 8 | 209 | 115 | 37767 | 4.40E-07 | 2.10E-05 | At4g23100, At4g39940, At4g14880, At1g74100, At3g45640, At3g19710, At1g62560, At5g27380 |
| GO:0006790 | P | sulfur metabolic process | 10 | 209 | 220 | 37767 | 6.70E-07 | 3.10E-05 | At4g23100, At4g39940, At5g09290, At4g14880, At1g74100, At2g44490, At3g45640, At3g19710, At1g62560, At5g27380 |
| GO:0009719 | P | response to endogenous stimulus | 21 | 209 | 1068 | 37767 | 7.50E-07 | 3.20E-05 | At2g38750, At3g45140, At4g23100, At5g24780, At1g32640, At5g67300, At3g45640, At4g23600, At2g33380, At1g55020 |
| GO:0019748 | P | secondary metabolic process | 14 | 209 | 489 | 37767 | 9.10E-07 | 3.70E-05 | At2g29490, At4g23100, At1g27140, At1g32640, At4g39940, At1g78370, At1g74100, At2g44490, At2g29440, At3g45640 |
| GO:0009695 | P | jasmonic acid biosynthetic process | 5 | 209 | 29 | 37767 | 1.20E-06 | 4.50E-05 | At3g25780, At1g55020, At1g17420, At3g45140, At2g06050 |
| GO:0031408 | P | oxylipin biosynthetic process | 5 | 209 | 32 | 37767 | 1.80E-06 | 6.70E-05 | At3g25780, At1g55020, At1g17420, At3g45140, At2g06050 |
| GO:0019760 | P | glucosinolate metabolic process | 6 | 209 | 62 | 37767 | 2.10E-06 | 6.90E-05 | At4g23100, At4g39940, At1g74100, At2g44490, At3g19710, At1g62560 |
| GO:0016143 | P | S-glycoside metabolic process | 6 | 209 | 62 | 37767 | 2.10E-06 | 6.90E-05 | At4g23100, At4g39940, At1g74100, At2g44490, At3g19710, At1g62560 |
| GO:0019757 | P | glycosinolate metabolic process | 6 | 209 | 62 | 37767 | 2.10E-06 | 6.90E-05 | At4g23100, At4g39940, At1g74100, At2g44490, At3g19710, At1g62560 |
| GO:0009694 | P | jasmonic acid metabolic process | 5 | 209 | 34 | 37767 | 2.40E-06 | 7.40E-05 | At3g25780, At1g55020, At1g17420, At3g45140, At2g06050 |
| GO:0016144 | P | S-glycoside biosynthetic process | 5 | 209 | 41 | 37767 | 5.50E-06 | 0.00015 | At4g39940, At3g19710, At1g74100, At1g62560, At4g23100 |
| GO:0019758 | P | glycosinolate biosynthetic process | 5 | 209 | 41 | 37767 | 5.50E-06 | 0.00015 | At4g39940, At3g19710, At1g74100, At1g62560, At4g23100 |
| GO:0006631 | P | fatty acid metabolic process | 9 | 209 | 225 | 37767 | 6.70E-06 | 0.00018 | At3g45140, At4g29010, At2g06050, At2g33380, At1g55020, At2g37700, At3g25780, At1g17420, At1g79750 |
| GO:0009414 | P | response to water deprivation | 9 | 209 | 229 | 37767 | 7.60E-06 | 0.0002 | At3g51920, At1g32640, At5g67300, At2g41430, At2g33380, At3g45140, At1g76180, At4g02380, At5g45340 |
| GO:0009628 | P | response to abiotic stimulus | 23 | 209 | 1471 | 37767 | 9.40E-06 | 0.00024 | At2g38750, At3g51920, At1g32640, At5g59820, At2g33380, At5g28540, At3g45640, At5g57560, At5g45340, At4g23100 |
| GO:0010038 | P | response to metal ion | 9 | 209 | 238 | 37767 | 1.00E-05 | 0.00025 | At4g23100, At4g14880, At2g41380, At5g67300, At5g24770, At1g07600, At5g37770, At3g20390, At3g51920 |
| GO:0009415 | P | response to water | 9 | 209 | 240 | 37767 | 1.10E-05 | 0.00025 | At3g51920, At1g32640, At5g67300, At2g41430, At2g33380, At3g45140, At1g76180, At4g02380, At5g45340 |
| GO:0009743 | P | response to carbohydrate stimulus | 9 | 209 | 240 | 37767 | 1.10E-05 | 0.00025 | At1g80840, At4g31800, At4g17230, At1g32640, At5g67300, At5g59820, At5g64660, At3g45640, At1g08930 |
| GO:0009617 | P | response to bacterium | 9 | 209 | 247 | 37767 | 1.40E-05 | 0.0003 | At4g23100, At1g80840, At4g31800, At5g06320, At2g41430, At2g25000, At1g55020, At2g44490, At3g45640 |
| GO:0009642 | P | response to light intensity | 6 | 209 | 90 | 37767 | 1.60E-05 | 0.00034 | At5g20230, At5g59820, At2g41430, At4g37270, At1g07600, At5g37770 |
| GO:0006633 | P | fatty acid biosynthetic process | 7 | 209 | 140 | 37767 | 1.80E-05 | 0.00038 | At3g45140, At2g06050, At2g37700, At1g55020, At3g25780, At1g17420, At1g79750 |
| GO:0006979 | P | response to oxidative stress | 10 | 209 | 332 | 37767 | 2.20E-05 | 0.00045 | At5g20230, At1g07890, At1g32640, At5g24770, At3g10920, At5g17000, At5g59820, At5g37770, At3g45640, At4g02380 |
| GO:0016137 | P | glycoside metabolic process | 6 | 209 | 104 | 37767 | 3.40E-05 | 0.00068 | At4g23100, At4g39940, At1g74100, At2g44490, At3g19710, At1g62560 |
| GO:0050832 | P | defense response to fungus | 6 | 209 | 108 | 37767 | 4.20E-05 | 0.00081 | At4g23100, At1g80840, At4g31800, At2g25000, At2g44490, At2g30020 |
| GO:0009737 | P | response to abscisic acid stimulus | 10 | 209 | 378 | 37767 | 6.40E-05 | 0.0012 | At2g38750, At3g51920, At1g32640, At5g67300, At4g23600, At2g33380, At1g55020, At1g76180, At3g45640, At5g37770 |
| GO:0042742 | P | defense response to bacterium | 7 | 209 | 177 | 37767 | 7.70E-05 | 0.0014 | At4g23100, At1g80840, At4g31800, At5g06320, At2g25000, At1g55020, At2g44490 |
| GO:0016138 | P | glycoside biosynthetic process | 5 | 209 | 79 | 37767 | 0.0001 | 0.0019 | At4g39940, At3g19710, At1g74100, At1g62560, At4g23100 |
| GO:0043436 | P | oxoacid metabolic process | 15 | 209 | 859 | 37767 | 0.00011 | 0.0019 | At3g45140, At4g29010, At4g14880, At1g79750, At5g17990, At4g23600, At2g33380, At1g55020, At1g25155, At2g37700 |
| GO:0006082 | P | organic acid metabolic process | 15 | 209 | 860 | 37767 | 0.00011 | 0.0019 | At3g45140, At4g29010, At4g14880, At1g79750, At5g17990, At4g23600, At2g33380, At1g55020, At1g25155, At2g37700 |
| GO:0019752 | P | carboxylic acid metabolic process | 15 | 209 | 859 | 37767 | 0.00011 | 0.0019 | At3g45140, At4g29010, At4g14880, At1g79750, At5g17990, At4g23600, At2g33380, At1g55020, At1g25155, At2g37700 |
| GO:0032787 | P | monocarboxylic acid metabolic process | 10 | 209 | 408 | 37767 | 0.00012 | 0.002 | At3g45140, At4g29010, At1g79750, At2g06050, At2g33380, At1g55020, At2g37700, At3g25780, At1g17420, At5g45340 |
| GO:0042180 | P | cellular ketone metabolic process | 15 | 209 | 882 | 37767 | 0.00015 | 0.0024 | At3g45140, At4g29010, At4g14880, At1g79750, At5g17990, At4g23600, At2g33380, At1g55020, At1g25155, At2g3770 |
| GO:0002376 | P | immune system process | 9 | 209 | 368 | 37767 | 0.00026 | 0.0041 | At4g23100, At1g80840, At4g31800, At5g06320, At1g55020, At1g74100, At2g44490, At1g29690, At5g37770 |
| GO:0006955 | P | immune response | 9 | 209 | 367 | 37767 | 0.00026 | 0.0041 | At4g23100, At1g80840, At4g31800, At5g06320, At1g55020, At1g74100, At2g44490, At1g29690, At5g37770 |
| GO:0009408 | P | response to heat | 6 | 209 | 161 | 37767 | 0.00034 | 0.0052 | At4g23100, At1g07890, At5g59820, At5g28540, At5g37770, At5g57560 |
| GO:0009266 | P | response to temperature stimulus | 10 | 209 | 485 | 37767 | 0.00045 | 0.0068 | At4g23100, At1g07890, At5g59820, At2g33380, At5g28540, At1g76180, At3g45640, At4g02380, At5g37770, At5g57560 |
| GO:0033554 | P | cellular response to stress | 9 | 209 | 399 | 37767 | 0.00047 | 0.0068 | At4g23100, At1g32640, At3g10920, At5g28540, At5g48850, At2g44490, At3g45640, At4g02380, At2g02990 |
| GO:0034637 | P | cellular carbohydrate biosynthetic process | 6 | 209 | 177 | 37767 | 0.00055 | 0.0078 | At4g23100, At4g32410, At4g39940, At1g74100, At3g19710, At1g62560 |
| GO:0006970 | P | response to osmotic stress | 9 | 209 | 408 | 37767 | 0.00055 | 0.0078 | At2g38750, At5g59820, At3g51920, At4g32410, At5g67300, At4g23600, At2g33380, At5g24770, At3g45640 |
| GO:0016053 | P | organic acid biosynthetic process | 9 | 209 | 417 | 37767 | 0.00064 | 0.0086 | At3g45140, At4g14880, At5g17990, At2g37700, At1g55020, At3g25780, At1g17420, At1g79750, At2g06050 |
| GO:0046394 | P | carboxylic acid biosynthetic process | 9 | 209 | 417 | 37767 | 0.00064 | 0.0086 | At3g45140, At4g14880, At5g17990, At2g37700, At1g55020, At3g25780, At1g17420, At1g79750, At2g06050 |
| GO:0006629 | P | lipid metabolic process | 13 | 209 | 841 | 37767 | 0.00097 | 0.013 | At3g45140, At3g08510, At4g29010, At4g19860, At2g06050, At2g33380, At1g55020, At1g02660, At2g37700, At3g25780 |
| GO:0009751 | P | response to salicylic acid stimulus | 6 | 209 | 200 | 37767 | 0.001 | 0.013 | At1g80840, At4g31800, At5g67300, At2g25000, At2g33380, At1g72260 |
| GO:0009409 | P | response to cold | 7 | 209 | 328 | 37767 | 0.0027 | 0.034 | At5g59820, At2g33380, At5g37770, At3g45640, At4g02380, At1g76180, At5g57560 |
| GO:0044255 | P | cellular lipid metabolic process | 10 | 209 | 618 | 37767 | 0.0027 | 0.034 | At3g45140, At4g29010, At1g79750, At2g06050, At2g33380, At1g55020, At2g37700, At3g25780, At1g17420, At5g45340 |
| GO:0046686 | P | response to cadmium ion | 5 | 209 | 178 | 37767 | 0.0035 | 0.044 | At3g20390, At5g67300, At4g23100, At4g14880, At2g41380 |
| GO:0003824 | F | catalytic activity | 82 | 209 | 9638 | 37767 | 1.00E-05 | 0.0019 | At4g14570, At1g21130, At3g08510, At5g19440, At1g11840, At4g29010, At4g39940, At5g58310, At5g17990, At2g34810 |
| GO:0005509 | F | calcium ion binding | 8 | 209 | 215 | 37767 | 3.60E-05 | 0.0033 | At2g38750, At3g51920, At2g33380, At2g46600, At1g76180, At5g37770, At4g27280, At1g73630 |
| GO:0016209 | F | antioxidant activity | 6 | 209 | 150 | 37767 | 0.00024 | 0.015 | At1g65980, At1g07890, At3g10920, At1g60740, At1g19550, At1g19570 |
| GO:0016491 | F | oxidoreductase activity | 19 | 209 | 1463 | 37767 | 0.00059 | 0.027 | At5g05600, At2g24270, At3g45140, At1g07890, At1g79750, At2g06050, At3g10920, At1g60740, At2g33380, At1g55020 |

**downregulated genes**

| | | | | | | | | | |
|---|---|---|---|---|---|---|---|---|---|
| GO:0044237 | P | cellular metabolic process | 26 | 47 | 8722 | 37767 | 1.90E-06 | 7.50E-05 | At4g20960, At2g02220, At5g54770, At1g06430, At1g70600, At1g55090, At1g08380, At3g24190, At2g34480, At1g12900 |
| GO:0015979 | P | photosynthesis | 5 | 47 | 162 | 37767 | 2.10E-06 | 7.50E-05 | At3g55800, At3g27690, At1g61520, At1g08380, At3g01440 |
| GO:0034641 | P | cellular nitrogen compound metabolic proce | 7 | 47 | 506 | 37767 | 3.20E-06 | 7.70E-05 | At4g20960, At1g55090, At5g54770, At4g27440, At4g38970, At5g17920, At2g43360 |
| GO:0008152 | P | metabolic process | 28 | 47 | 10614 | 37767 | 6.60E-06 | 0.00012 | At4g20960, At2g02220, At5g54770, At1g06430, At4g20760, At1g70600, At1g55090, At1g08380, At3g24190, At2g34480 |
| GO:0044271 | P | cellular nitrogen compound biosynthetic pro | 5 | 47 | 394 | 37767 | 0.00014 | 0.0018 | At4g20960, At2g43360, At5g54770, At4g27440, At5g17920 |
| GO:0009987 | P | cellular process | 27 | 47 | 11684 | 37767 | 0.00015 | 0.0018 | At4g20960, At2g02220, At5g54770, At1g06430, At1g70600, At1g55090, At1g08380, At3g24190, At2g34480, At1g12900 |
| GO:0001883 | F | purine nucleoside binding | 7 | 47 | 1465 | 37767 | 0.0021 | 0.014 | At1g55090, At2g02220, At3g24190, At3g28540, At3g03960, At4g28070, At2g45340 |
| GO:0001882 | F | nucleoside binding | 7 | 47 | 1465 | 37767 | 0.0021 | 0.014 | At1g55090, At2g02220, At3g24190, At3g28540, At3g03960, At4g28070, At2g45340 |
| GO:0000166 | F | nucleotide binding | 9 | 47 | 2267 | 37767 | 0.0017 | 0.014 | At1g55090, At2g02220, At3g24190, At2g45340, At3g28540, At3g03960, At4g28070, At5g04810, At1g43190 |
| GO:0005524 | F | ATP binding | 7 | 47 | 1377 | 37767 | 0.0015 | 0.014 | At1g55090, At2g02220, At3g24190, At3g28540, At3g03960, At4g28070, At2g45340 |
| GO:0003824 | F | catalytic activity | 22 | 47 | 9638 | 37767 | 0.0013 | 0.014 | At4g20960, At4g24780, At2g02220, At1g06430, At3g26060, At4g27440, At5g19220, At1g55090, At3g24190, At3g28540 |
| GO:0030554 | F | adenyl nucleotide binding | 7 | 47 | 1465 | 37767 | 0.0021 | 0.014 | At1g55090, At2g02220, At3g24190, At3g28540, At3g03960, At4g28070, At2g45340 |
| GO:0032559 | F | adenyl ribonucleotide binding | 7 | 47 | 1384 | 37767 | 0.0016 | 0.014 | At1g55090, At2g02220, At3g24190, At3g28540, At3g03960, At4g28070, At2g45340 |
| GO:0032555 | F | purine ribonucleotide binding | 7 | 47 | 1631 | 37767 | 0.0039 | 0.02 | At1g55090, At2g02220, At3g24190, At3g28540, At3g03960, At4g28070, At2g45340 |
| GO:0032553 | F | ribonucleotide binding | 7 | 47 | 1631 | 37767 | 0.0039 | 0.02 | At1g55090, At2g02220, At3g24190, At3g28540, At3g03960, At4g28070, At2g45340 |
| GO:0017076 | F | purine nucleotide binding | 7 | 47 | 1713 | 37767 | 0.0051 | 0.023 | At1g55090, At2g02220, At3g24190, At3g28540, At3g03960, At4g28070, At2g45340 |
| GO:0044435 | C | plastid part | 10 | 47 | 867 | 37767 | 1.00E-07 | 4.00E-06 | At3g63140, At2g47400, At5g54770, At5g17670, At1g61520, At3g26060, At3g01440, At4g38970, At5g38410, At5g13630 |
| GO:0009507 | C | chloroplast | 16 | 47 | 2740 | 37767 | 1.00E-07 | 4.00E-06 | At4g20960, At3g63140, At5g17670, At2g47400, At3g24190, At5g54770, At4g27440, At1g06430, At1g61520, At3g26060 |
| GO:0009536 | C | plastid | 16 | 47 | 2965 | 37767 | 3.00E-07 | 6.40E-06 | At4g20960, At3g63140, At5g17670, At2g47400, At3g24190, At5g54770, At4g27440, At1g06430, At1g61520, At3g26060 |
| GO:0044434 | C | chloroplast part | 9 | 47 | 746 | 37767 | 3.30E-07 | 6.40E-06 | At3g63140, At2g47400, At5g17670, At1g61520, At3g26060, At3g01440, At4g38970, At5g38410, At5g13630 |
| GO:0044446 | C | intracellular organelle part | 14 | 47 | 2561 | 37767 | 1.80E-06 | 2.00E-05 | At3g63140, At2g47400, At5g13930, At5g54770, At2g34480, At5g17670, At1g61520, At3g26060, At3g01440, At4g38970 |
| GO:0044444 | C | cytoplasmic part | 22 | 47 | 6289 | 37767 | 1.50E-06 | 2.00E-05 | At4g20960, At5g54770, At1g06430, At3g26060, At1g70600, At5g46580, At3g24190, At2g34480, At5g13930, At5g04810 |
| GO:0044422 | C | organelle part | 14 | 47 | 2562 | 37767 | 1.80E-06 | 2.00E-05 | At3g63140, At2g47400, At5g13930, At5g54770, At2g34480, At5g17670, At1g61520, At3g26060, At3g01440, At4g38970 |
| GO:0005622 | C | intracellular | 27 | 47 | 9671 | 37767 | 3.70E-06 | 3.60E-05 | At4g20960, At5g54770, At1g06430, At3g26060, At1g70600, At5g46580, At3g15680, At1g08380, At3g24190, At2g34480 |
| GO:0005737 | C | cytoplasm | 22 | 47 | 6822 | 37767 | 6.00E-06 | 5.10E-05 | At4g20960, At5g54770, At1g06430, At3g26060, At1g70600, At5g46580, At3g24190, At2g34480, At5g13930, At5g04810 |
| GO:0044424 | C | intracellular part | 26 | 47 | 9302 | 37767 | 6.70E-06 | 5.20E-05 | At4g20960, At5g54770, At1g06430, At3g26060, At1g70600, At5g46580, At1g08380, At3g24190, At2g34480, At5g13930 |
| GO:0043226 | C | organelle | 22 | 47 | 8155 | 37767 | 0.00011 | 0.0007 | At4g20960, At5g54770, At1g06430, At3g26060, At1g70600, At5g46580, At3g24190, At2g34480, At5g13930, At5g04810 |
| GO:0043229 | C | intracellular organelle | 22 | 47 | 8149 | 37767 | 0.00011 | 0.0007 | At4g20960, At5g54770, At1g06430, At3g26060, At1g70600, At5g46580, At3g24190, At2g34480, At5g13930, At5g04810 |
| GO:0005623 | C | cell | 31 | 47 | 15217 | 37767 | 0.00034 | 0.0018 | At4g20960, At4g24780, At5g54770, At1g06430, At3g26060, At1g70600, At5g46580, At3g15680, At1g08380, At2g45340 |
| GO:0044464 | C | cell part | 31 | 47 | 15217 | 37767 | 0.00034 | 0.0018 | At4g20960, At4g24780, At5g54770, At1g06430, At3g26060, At1g70600, At5g46580, At3g15680, At1g08380, At2g45340 |
| GO:0032991 | C | macromolecular complex | 9 | 47 | 2180 | 37767 | 0.0013 | 0.0067 | At1g08380, At5g38410, At3g27690, At2g34480, At1g61520, At3g01440, At2g07696, At1g70600, At5g13630 |
| GO:0043227 | C | membrane-bounded organelle | 18 | 47 | 7622 | 37767 | 0.0033 | 0.015 | At4g20960, At1g43190, At3g63140, At5g17670, At2g47400, At3g24190, At5g54770, At4g27440, At1g06430, At1g61520 |
| GO:0043231 | C | intracellular membrane-bounded organelle | 18 | 47 | 7615 | 37767 | 0.0032 | 0.015 | At4g20960, At1g43190, At3g63140, At5g17670, At2g47400, At3g24190, At5g54770, At4g27440, At1g06430, At1g61520 |
| GO:0043234 | C | protein complex | 6 | 47 | 1443 | 37767 | 0.0088 | 0.036 | At1g08380, At5g38410, At3g27690, At1g61520, At3g01440, At5g13630 |
| GO:0043232 | C | intracellular non-membrane-bounded organe | 5 | 47 | 1040 | 37767 | 0.0094 | 0.036 | At1g70600, At5g41790, At2g34480, At5g38410, At2g07696 |
| GO:0043228 | C | non-membrane-bounded organelle | 5 | 47 | 1040 | 37767 | 0.0094 | 0.036 | At1g70600, At5g41790, At2g34480, At5g38410, At2g07696 |
