# Supplementary material for: Combined biotic stresses trigger similar transcriptomic responses but contrasting resistance against a chewing herbivore in Brassica nigra
Source: BMC Plant Biol. 2017 Jul 17;17:127. doi: 10.1186/s12870-017-1074-7 (PMC5513356; doi:10.1186/s12870-017-1074-7)
Supplement: Supplementary file 3 — Expression of the top-50 upregulated genes in response to P. brassicae feeding and combined stresses. The highest significantly upregulated genes (log2 > 0.585, P < 0.05) were extracted from microarray data (orange bars) and plotted with values from combined stresses. (A) Egg extract/P. brassicae larvae (yellow bars), (B) Xanthomonas campestris pv. raphani/P. brassicae larvae (green bars), and (C) Brevicoryne brassicae/P. brassicae larvae (blue bars). Significant differences between single and combined stress are indicated (Student’s t-test, ***P < 0.001, **P < 0.01, *P < 0.05). (PDF 1871 kb) [file 12870_2017_1074_MOESM3_ESM.pdf]

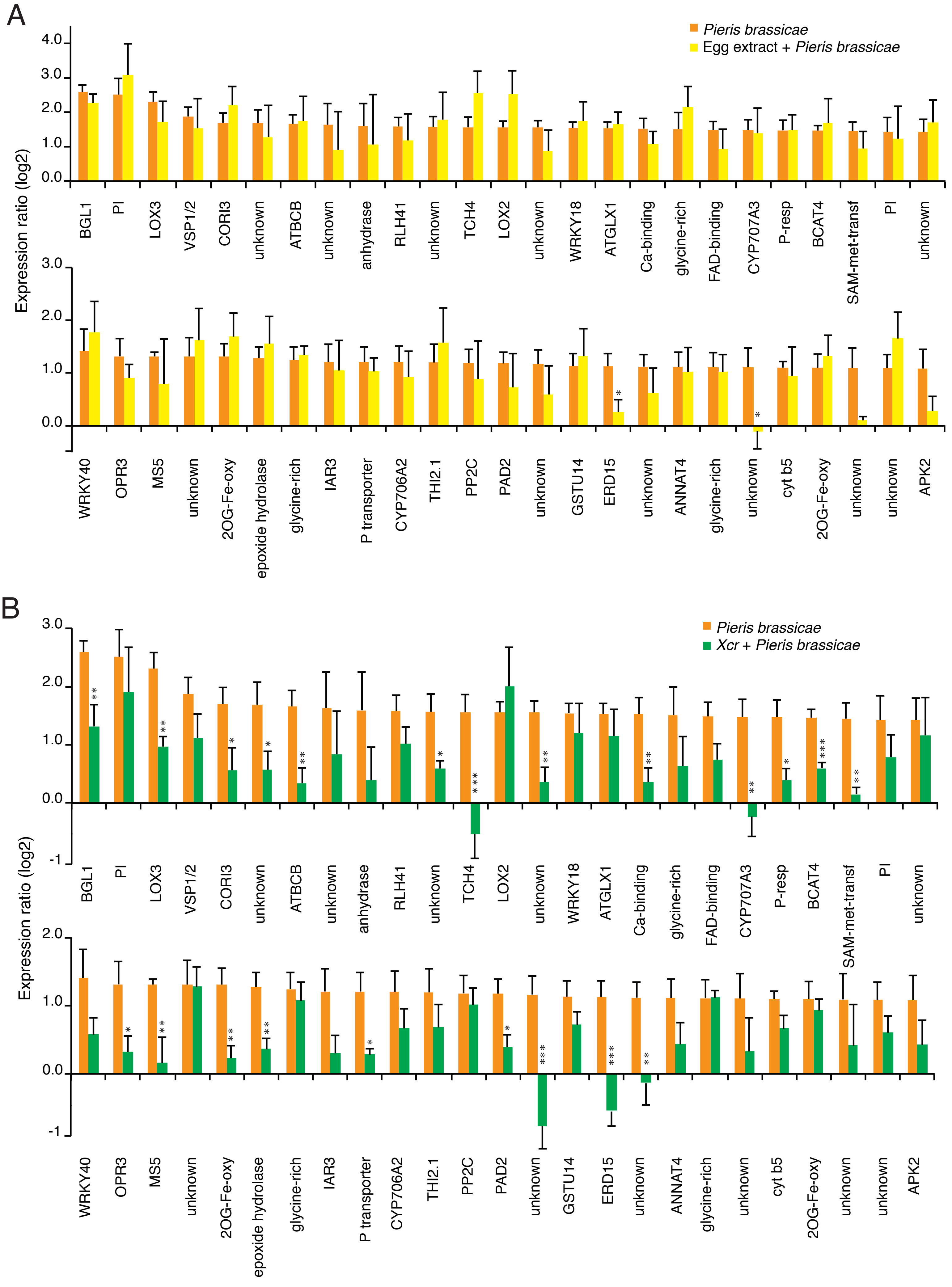
A
Pieris brassicae
Egg extract + Pieris brassicae
Expression ratio (log2)
4.0
3.0
2.0
1.0
0.0
BGL1
PI
LOX3
VSP1/2
CORI3
unknown
ATBCB
unknown
anhydrase
RLH41
unknown
TCH4
LOX2
unknown
WRKY18
ATGLX1
Ca-binding
glycine-rich
FAD-binding
CYP707A3
P-resp
BCAT4
SAM-met-transf
PI
unknown
3.0
2.0
1.0
0.0
WRKY40
OPR3
MS5
unknown
2OG-Fe-oxy
epoxide hydrolase
glycine-rich
IAR3
P transporter
CYP706A2
THI2.1
PP2C
PAD2
unknown
GSTU14
ERD15
unknown
ANNAT4
glycine-rich
unknown
cyt b5
2OG-Fe-oxy
unknown
unknown
APK2
B
Pieris brassicae
Xcr + Pieris brassicae
Expression ratio (log2)
3.0
2.0
1.0
0.0
-1
BGL1
PI
LOX3
VSP1/2
CORI3
unknown
ATBCB
unknown
anhydrase
RLH41
unknown
TCH4
LOX2
unknown
WRKY18
ATGLX1
Ca-binding
glycine-rich
FAD-binding
CYP707A3
P-resp
BCAT4
SAM-met-transf
PI
unknown
2.0
1.0
0.0
-1
WRKY40
OPR3
MS5
unknown
2OG-Fe-oxy
epoxide hydrolase
glycine-rich
IAR3
P transporter
CYP706A2
THI2.1
PP2C
PAD2
unknown
GSTU14
ERD15
unknown
ANNAT4
glycine-rich
unknown
cyt b5
2OG-Fe-oxy
unknown
unknown
APK2

Fig. S1

Continued

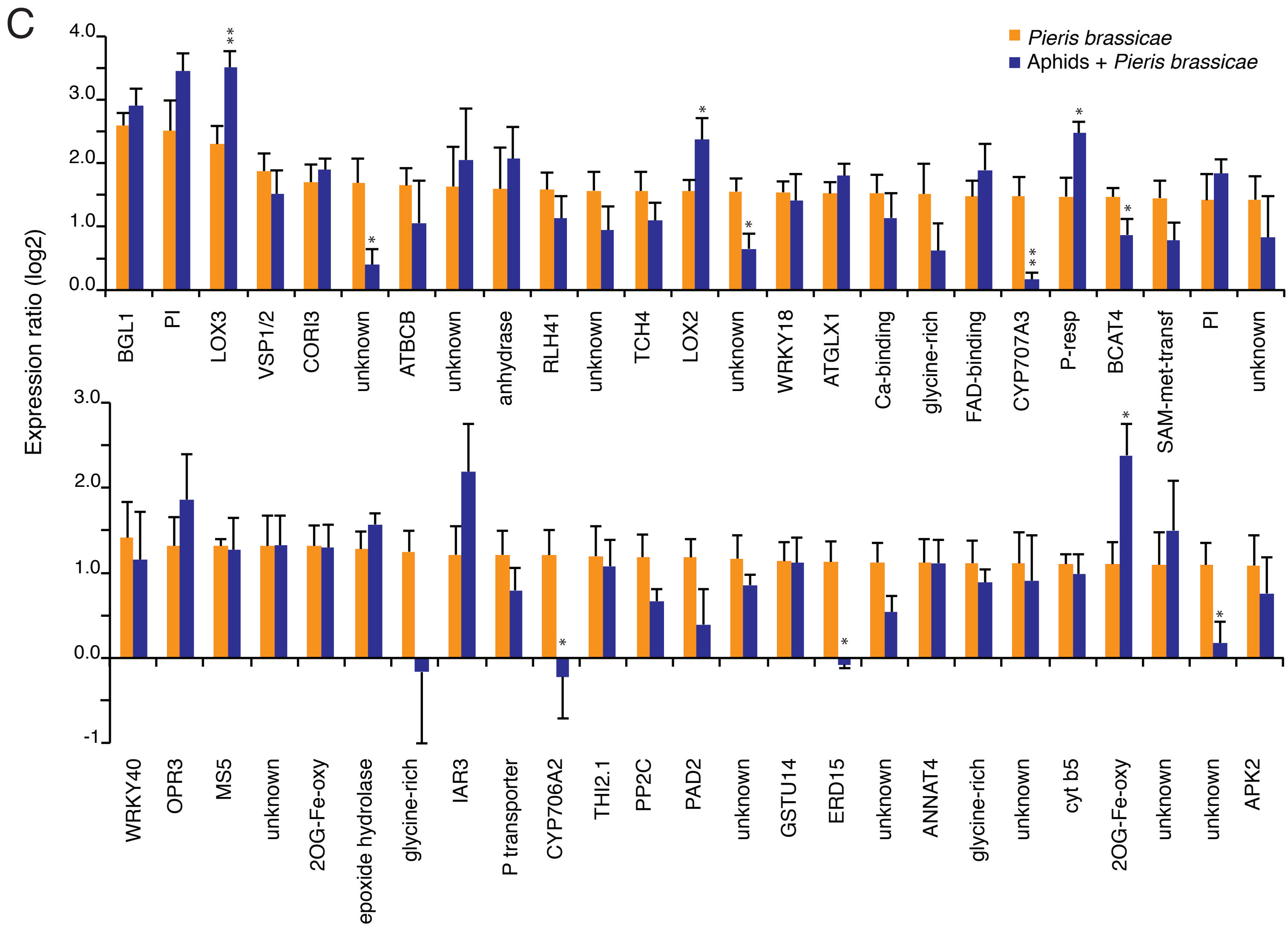

Fig. S1. Expression of the top-50 upregulated genes in response to *P. brassicae* feeding and combined stresses. The highest significantly upregulated genes ($\log_2 > 0.585$, $P < 0.05$) were extracted from microarray data (orange bars) and plotted with values from combined stresses. (A) Egg extract /*P. brassicae* larvae (yellow bars), (B) *Xanthomonas campestris* pv. *raphani* /*P. brassicae* larvae (green bars), and (C) *Brevicoryne brassicae*/*P. brassicae* larvae (blue bars). Significant differences between single and combined stress are indicated (Student's *t*-test, ***P<0.001, **P< 0.01, *P< 0.05).
