# Supplementary material for: Combined biotic stresses trigger similar transcriptomic responses but contrasting resistance against a chewing herbivore in Brassica nigra
Source: BMC Plant Biol. 2017 Jul 17;17:127. doi: 10.1186/s12870-017-1074-7 (PMC5513356; doi:10.1186/s12870-017-1074-7)
Supplement: Supplementary file 4 — Expression of the top-50 downregulated genes in response to P. brassicae feeding and combined stresses. The highest significantly downregulated genes (log2 < −0.585, P < 0.05) were extracted from microarray data (orange bars) and plotted with values from combined stresses. (A) Egg extract/P. brassicae larvae (yellow bars), (B) Xanthomonas campestris pv. raphani/P. brassicae larvae (green bars), and (C) Brevicoryne brassicae/P. brassicae larvae (blue bars). Significant differences between single and combined stress are indicated (Student’s t-test, ***P < 0.001, **P < 0.01, *P < 0.05). (PDF 1875 kb) [file 12870_2017_1074_MOESM4_ESM.pdf]

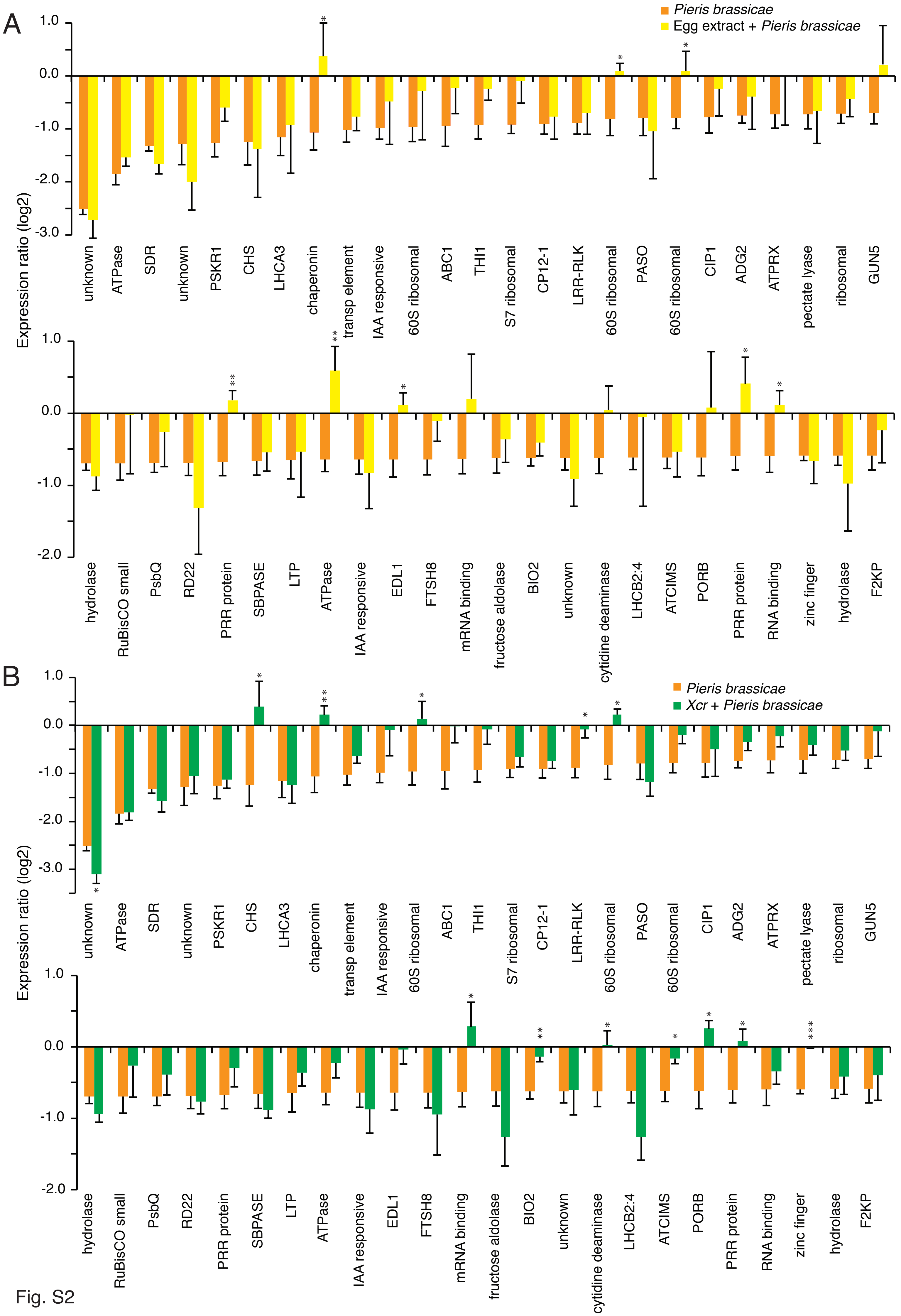

A
Pieris brassicae
Egg extract + Pieris brassicae
Expression ratio (log2)
1.0
0.0
-1.0
-2.0
-3.0
unknown
ATPase
SDR
unknown
PSKR1
CHS
LHCA3
chaperonin
transp element
IAA responsive
60S ribosomal
ABC1
THI1
S7 ribosomal
CP12-1
LRR-RLK
60S ribosomal
PASO
60S ribosomal
CIP1
ADG2
ATPRX
pectate lyase
ribosomal
GUN5
hydrolase
RuBisCO small
PsbQ
RD22
PRR protein
SBPASE
LTP
ATPase
IAA responsive
EDL1
FTSH8
mRNA binding
fructose aldolase
BIO2
unknown
cytidine deaminase
LHCB2:4
ATCIMS
PORB
PRR protein
RNA binding
zinc finger
hydrolase
F2KP
B
Pieris brassicae
Xcr + Pieris brassicae
Expression ratio (log2)

Fig. S2

Continued

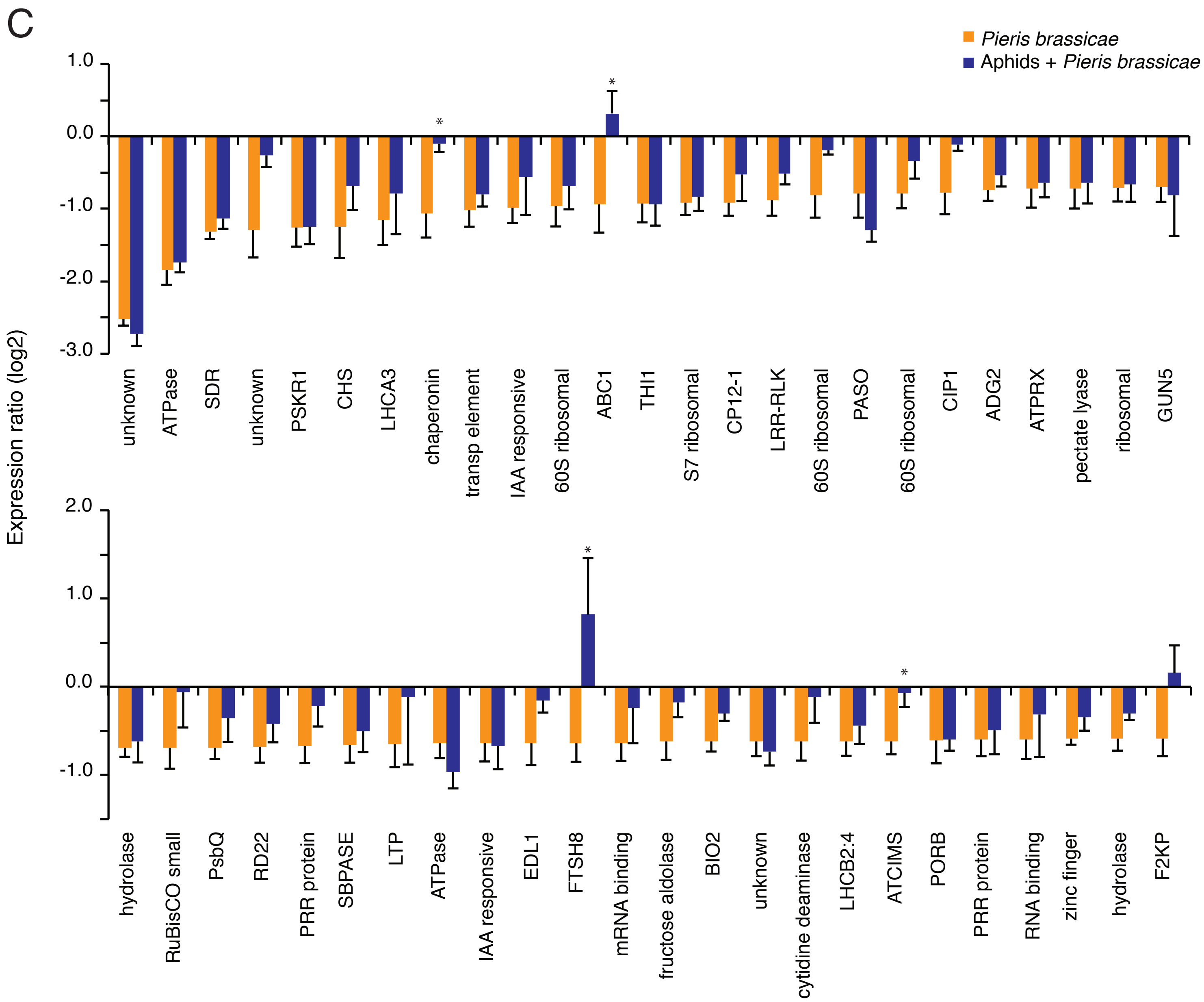

Fig. S2. Expression of the top-50 downregulated genes in response to *P. brassicae* feeding and combined stresses. The highest significantly downregulated genes ($\log_2 < -0.585$, $P < 0.05$) were extracted from microarray data (orange bars) and plotted with values from combined stresses. (A) Egg extract/*P. brassicae* larvae (yellow bars), (B) *Xanthomonas campestris* pv. *raphani* /*P. brassicae* larvae (green bars), and (C) *Brevicoryne brassicae*/*P. brassicae* larvae (blue bars). Significant differences between single and combined stress are indicated (Student's *t*-test, ****P*<0.001, ***P*< 0.01, **P*< 0.05).
