# Supplementary material for: Combined biotic stresses trigger similar transcriptomic responses but contrasting resistance against a chewing herbivore in Brassica nigra
Source: BMC Plant Biol. 2017 Jul 17;17:127. doi: 10.1186/s12870-017-1074-7 (PMC5513356; doi:10.1186/s12870-017-1074-7)
Supplement: Supplementary file 5 — GO analysis of genes specifically upregulated by combined stress. GO terms significantly enriched with each combined stress are shown separately. Length of the bars shows the percentage of regulated genes in the respective GO categories. (PDF 951 kb) [file 12870_2017_1074_MOESM5_ESM.pdf]

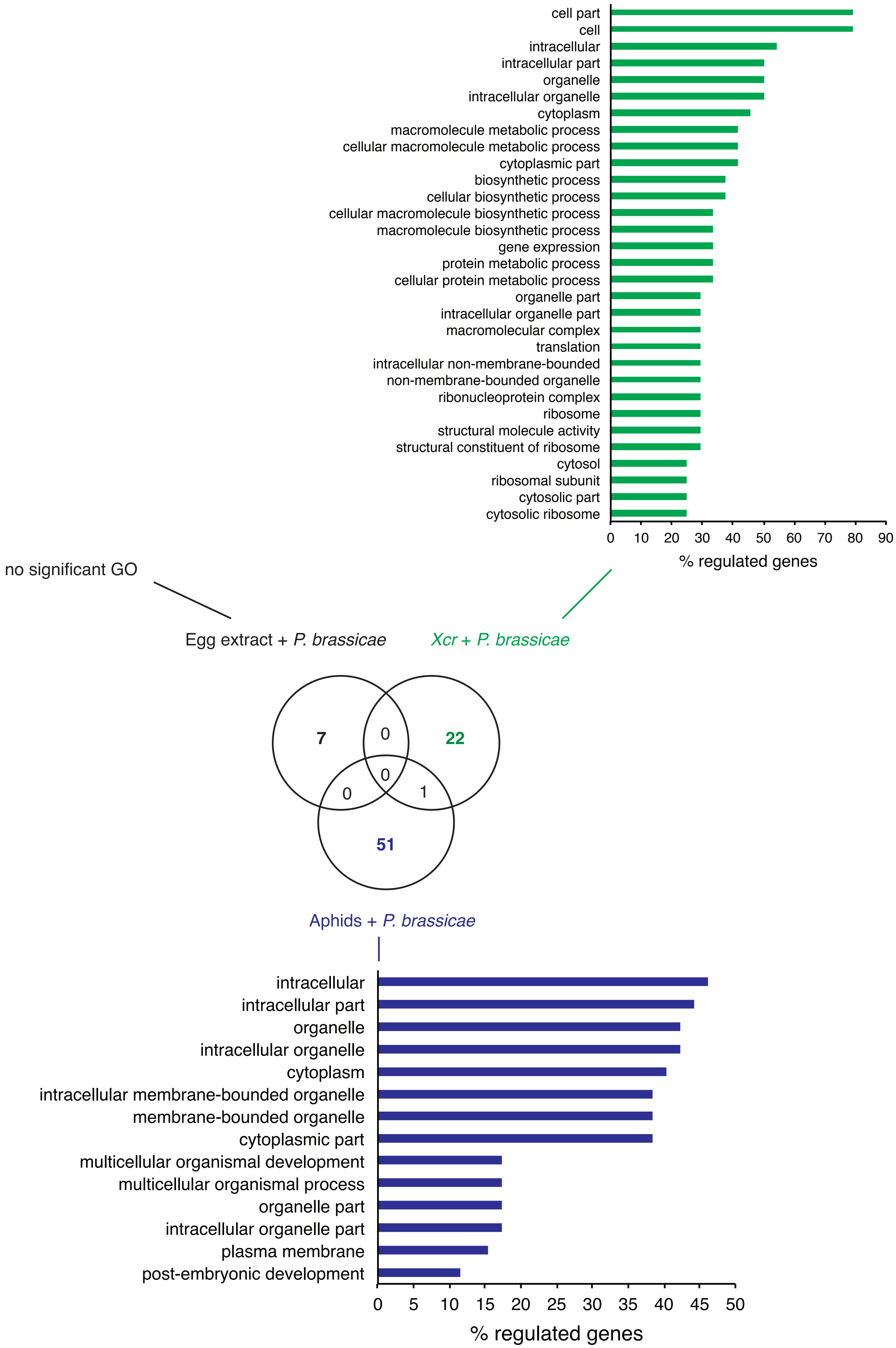

Fig. S3. GO analysis of genes specifically upregulated by combined stress. GO terms significantly enriched with each combined stress are shown separately. Length of the bars shows the percentage of regulated genes in the respective GO categories.
