# Supplementary material for: Combined biotic stresses trigger similar transcriptomic responses but contrasting resistance against a chewing herbivore in Brassica nigra
Source: BMC Plant Biol. 2017 Jul 17;17:127. doi: 10.1186/s12870-017-1074-7 (PMC5513356; doi:10.1186/s12870-017-1074-7)
Supplement: Supplementary file 6 — GO analysis of genes specifically downregulated by combined stress. GO analysis of genes specifically downregulated by combined stress. GO terms significantly enriched with each combined stress are shown separately. Length of the bars shows the percentage of regulated genes in the respective GO categories. (PDF 918 kb) [file 12870_2017_1074_MOESM6_ESM.pdf]

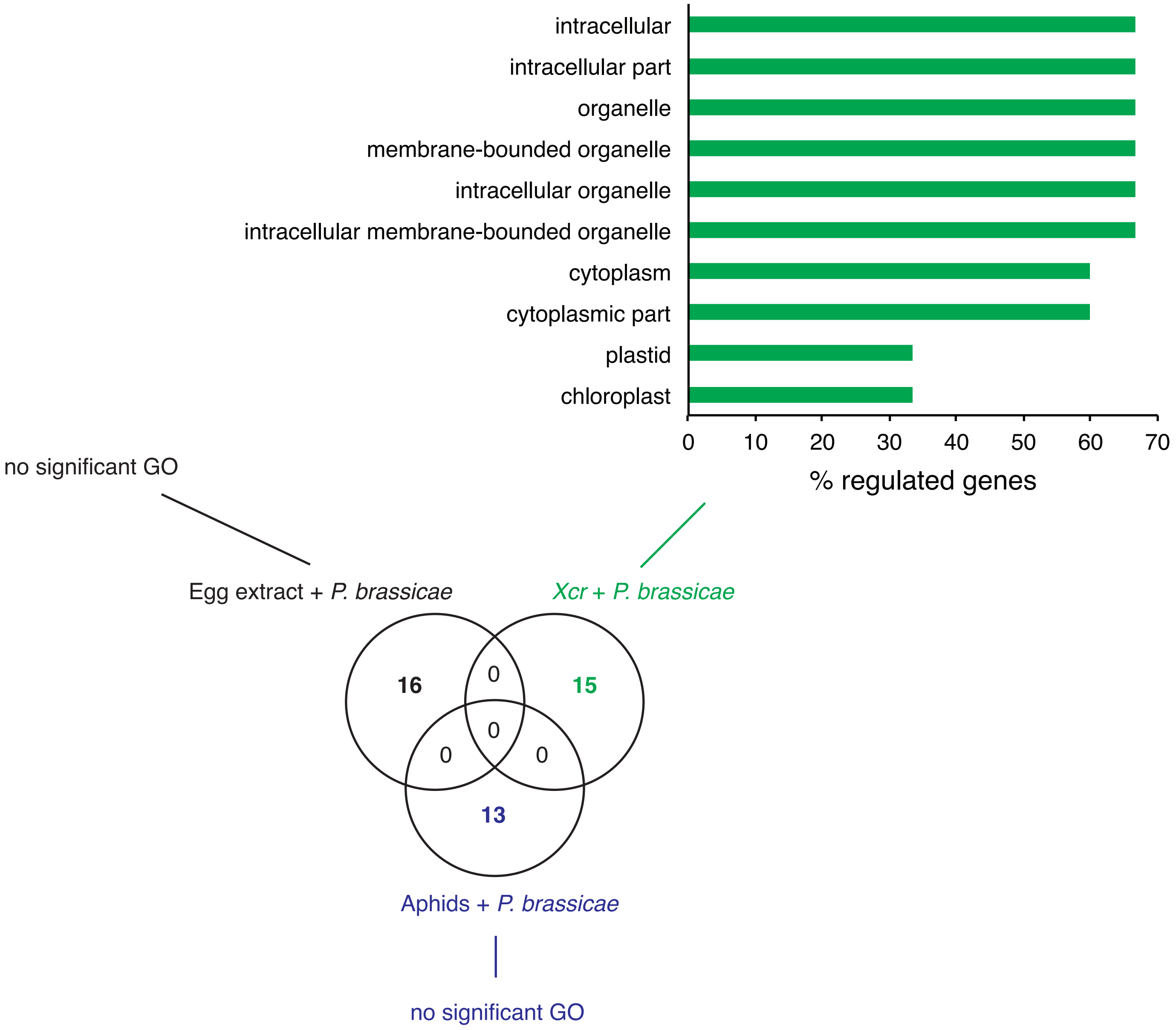

Fig. S4. GO analysis of genes specifically downregulated by combined stress. GO terms significantly enriched with each combined stress are shown separately. Length of the bars shows the percentage of regulated genes in the respective GO categories.
