# Supplementary material for: Combined biotic stresses trigger similar transcriptomic responses but contrasting resistance against a chewing herbivore in Brassica nigra
Source: BMC Plant Biol. 2017 Jul 17;17:127. doi: 10.1186/s12870-017-1074-7 (PMC5513356; doi:10.1186/s12870-017-1074-7)
Supplement: Supplementary file 7 — Quantification of defense signals and glucosinolates. (A) Experimental design. (B) Quantification of jasmonic acid (JA), salicylic acid (SA) and total glucosinolates (GS) in single and combined stress in whole 5-week-old B. nigra leaves. Plants were pretreated for 3 days with P. brassicae egg extract (EE) or Xanthomonas campestris pv. raphani (Xcr) and further challenged with P. brassicae (P) larvae for 24 h. Controls (CTL) consisted of untreated plants or plants exposed to a single treatment. Values (± SE) are the mean of three independent experiments. Letters indicate significant difference between treatments (two-way ANOVA followed by Tukey’s honest significant difference test, P < 0.05). (PDF 1050 kb) [file 12870_2017_1074_MOESM7_ESM.pdf]

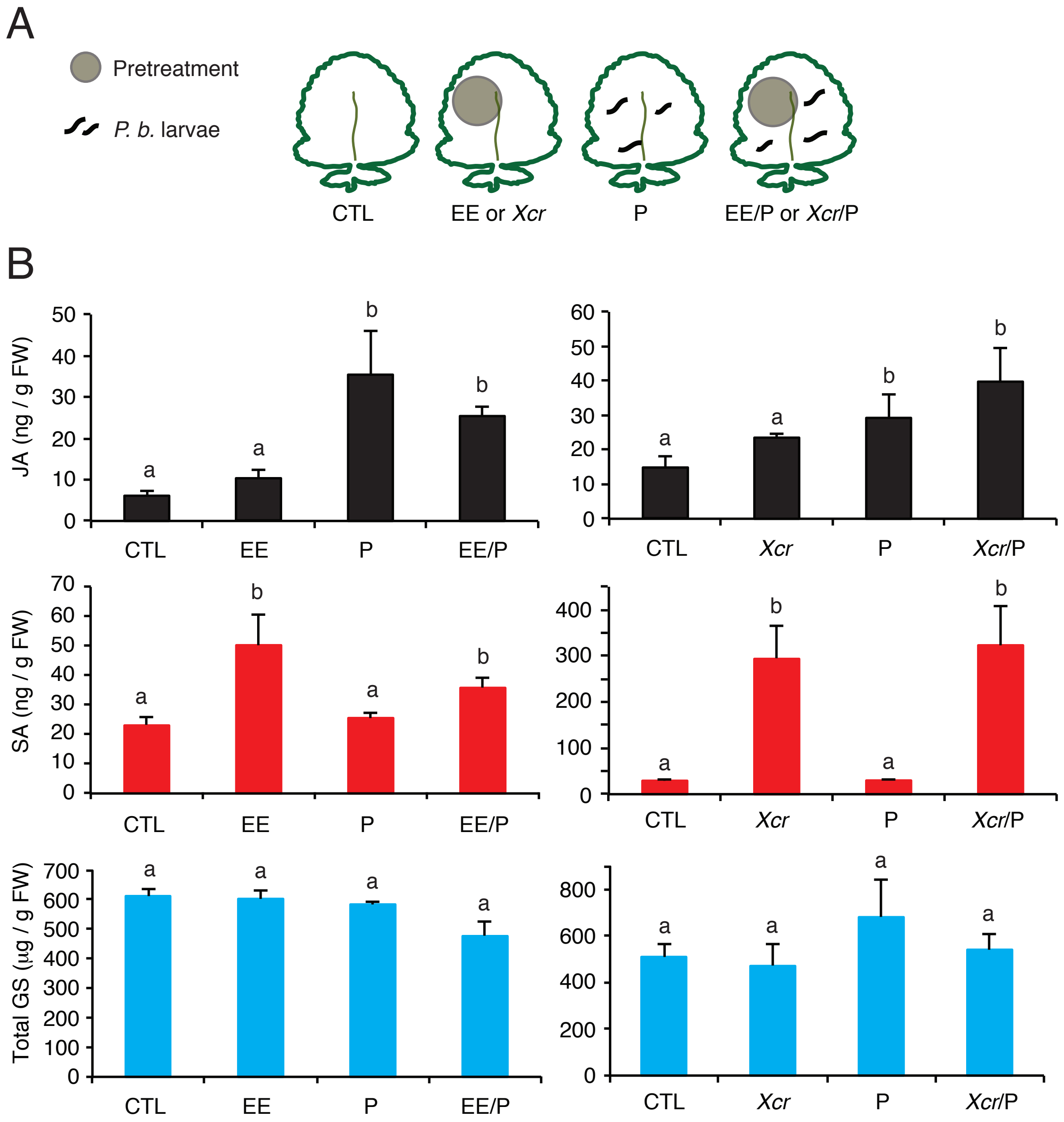

Fig. S5. Quantification of defense signals and glucosinolates. (A) Experimental design. (B) Quantification of jasmonic acid (JA), salicylic acid (SA) and total glucosinolates (GS) in single and combined stress in whole 5-week-old *B. nigra* leaves. Plants were pretreated for three days with *P. brassicae* egg extract (EE) or *Xanthomonas campestris* pv. *raphani* (*Xcr*) and further challenged with *P. brassicae* (P) larvae for 24 h. Controls (CTL) consisted of untreated plants or plants exposed to a single treatment. Values (± SE) are the mean of three independent experiments. Letters indicate significant difference between treatments (two-way ANOVA followed by Tukey's honest significant difference test, $P<0.05$).
