# Supplementary material for: Combined biotic stresses trigger similar transcriptomic responses but contrasting resistance against a chewing herbivore in Brassica nigra
Source: BMC Plant Biol. 2017 Jul 17;17:127. doi: 10.1186/s12870-017-1074-7 (PMC5513356; doi:10.1186/s12870-017-1074-7)
Supplement: Supplementary file 8 — Glucosinolate content in B. nigra leaves. (PDF 37 kb) [file 12870_2017_1074_MOESM8_ESM.pdf]

**Table S3.** Glucosinolate content in *B. nigra* leaves

| | Sample | Mean (μg/gFW) | SE | % of total | Sample | Mean (μg/gFW) | SE | % of total |
|---|---|---|---|---|---|---|---|---|
| Sinigrin | CTL | 1133.56 | 275.22 | 96.36 | CTL | 600.82 | 40.58 | 95.04 |
| | Egg extract (EE) | 751.69 | 16.94 | 94.80 | Xcc | 684.16 | 82.47 | 91.45 |
| | Pieris brassicae | 967.18 | 119.75 | 95.99 | Pieris | 576.15 | 22.36 | 92.91 |
| | CTL local | 615.56 | 144.10 | 94.66 | CTL local | 828.73 | 81.89 | 96.16 |
| | EE + Pieris brassicae | 546.20 | 122.66 | 90.97 | Xcc + Pieris | 701.42 | 84.57 | 92.05 |
| | EE distal | 537.74 | 76.71 | 92.07 | Xcc distal | 706.24 | 133.30 | 93.47 |
| | Pieris brassicae distal | 533.50 | 114.16 | 91.48 | Pieris distal | 722.68 | 167.56 | 94.33 |
| Glucoiberin | CTL | 0.48 | 0.10 | | CTL | 0.28 | 0.03 | |
| | Egg extract (EE) | 0.46 | 0.07 | | Xcc | 0.21 | 0.10 | |
| | Pieris brassicae | 0.56 | 0.11 | | Pieris | 0.56 | 0.16 | |
| | CTL local | 0.36 | 0.05 | | CTL local | 0.35 | 0.19 | |
| | EE + Pieris brassicae | 0.71 | 0.11 | | Xcc + Pieris | 0.39 | 0.08 | |
| | EE distal | 0.32 | 0.17 | | Xcc distal | 0.35 | 0.09 | |
| | Pieris brassicae distal | 0.40 | 0.08 | | Pieris distal | 0.18 | 0.10 | |
| Unknown A | CTL | 0.07 | 0.04 | | CTL | 0.09 | 0.05 | |
| | Egg extract (EE) | 0.03 | 0.02 | | Xcc | 0.06 | 0.03 | |
| | Pieris brassicae | 0.07 | 0.03 | | Pieris | 0.10 | 0.04 | |
| | CTL local | 0.04 | 0.03 | | CTL local | 0.06 | 0.04 | |
| | EE + Pieris brassicae | 0.07 | 0.01 | | Xcc + Pieris | 0.04 | 0.04 | |
| | EE distal | 0.08 | 0.02 | | Xcc distal | 0.11 | 0.04 | |
| | Pieris brassicae distal | 0.02 | 0.01 | | Pieris distal | 0.04 | 0.04 | |
| Sinalbin | CTL | 10.40 | 0.09 | | CTL | 10.46 | 0.22 | |
| | Egg extract (EE) | 11.10 | 0.62 | | Xcc | 10.73 | 0.36 | |
| | Pieris brassicae | 10.64 | 0.16 | | Pieris | 11.28 | 0.33 | |
| | CTL local | 10.97 | 0.62 | | CTL local | 11.56 | 0.58 | |
| | EE + Pieris brassicae | 10.28 | 0.08 | | Xcc + Pieris | 17.47 | 4.54 | |
| | EE distal | 11.72 | 0.42 | | Xcc distal | 12.07 | 0.74 | |
| | Pieris brassicae distal | 10.93 | 0.51 | | Pieris distal | 13.25 | 1.39 | |
| Gluconapin | CTL | 4.17 | 1.21 | | CTL | 2.01 | 0.17 | |
| | Egg extract (EE) | 2.59 | 0.30 | | Xcc | 2.16 | 0.27 | |
| | Pieris brassicae | 2.64 | 0.56 | | Pieris | 1.72 | 0.32 | |
| | CTL local | 2.32 | 0.82 | | CTL local | 2.53 | 0.57 | |
| | EE + Pieris brassicae | 2.11 | 0.73 | | Xcc + Pieris | 1.84 | 0.86 | |
| | EE distal | 1.71 | 0.38 | | Xcc distal | 2.59 | 0.60 | |
| | Pieris brassicae distal | 1.79 | 0.82 | | Pieris distal | 2.67 | 0.60 | |
| Unknown D | CTL | 7.27 | 2.51 | | CTL | 2.36 | 0.50 | |
| | Egg extract (EE) | 4.92 | 0.90 | | Xcc | 4.78 | 1.07 | |
| | Pieris brassicae | 5.04 | 2.18 | | Pieris | 4.51 | 1.73 | |
| | CTL local | 4.18 | 0.42 | | CTL local | 2.73 | 0.32 | |
| | EE + Pieris brassicae | 8.37 | 1.22 | | Xcc + Pieris | 6.14 | 1.10 | |
| | EE distal | 6.69 | 1.77 | | Xcc distal | 3.97 | 0.41 | |
| | Pieris brassicae distal | 7.35 | 1.79 | | Pieris distal | 4.26 | 0.32 | |
| Glucotropeolin | CTL | 0.51 | 0.16 | | CTL | 0.39 | 0.07 | |
| | Egg extract (EE) | 0.56 | 0.11 | | Xcc | 0.50 | 0.17 | |
| | Pieris brassicae | 0.42 | 0.26 | | Pieris | 0.56 | 0.04 | |
| | CTL local | 0.28 | 0.10 | | CTL local | 0.64 | 0.24 | |
| | EE + Pieris brassicae | 0.59 | 0.17 | | Xcc + Pieris | 0.93 | 0.35 | |
| | EE distal | 0.45 | 0.05 | | Xcc distal | 0.66 | 0.17 | |
| | Pieris brassicae distal | 0.45 | 0.02 | | Pieris distal | 0.55 | 0.14 | |
| Glucobrassicin | CTL | 5.66 | 1.39 | | CTL | 3.82 | 1.08 | |
| | Egg extract (EE) | 8.78 | 2.71 | | Xcc | 23.66 | 2.86 | |
| | Pieris brassicae | 6.09 | 2.53 | | Pieris | 6.67 | 2.08 | |
| | CTL local | 4.81 | 1.04 | | CTL local | 3.11 | 0.51 | |
| | EE + Pieris brassicae | 17.91 | 3.61 | | Xcc + Pieris | 17.65 | 4.44 | |
| | EE distal | 13.21 | 2.80 | | Xcc distal | 13.88 | 3.80 | |
| | Pieris brassicae distal | 16.44 | 4.47 | | Pieris distal | 7.79 | 1.84 | |
| Hydroxyglucobrassicin | CTL | 0.60 | 0.19 | | CTL | 0.20 | 0.01 | |
| | Egg extract (EE) | 0.58 | 0.14 | | Xcc | 1.27 | 0.30 | |
| | Pieris brassicae | 0.51 | 0.17 | | Pieris | 0.32 | 0.25 | |
| | CTL local | 0.27 | 0.17 | | CTL local | 0.28 | 0.13 | |
| | EE + Pieris brassicae | 1.63 | 0.42 | | Xcc + Pieris | 1.08 | 0.04 | |
| | EE distal | 1.22 | 0.42 | | Xcc distal | 0.39 | 0.23 | |
| | Pieris brassicae distal | 1.38 | 0.64 | | Pieris distal | 0.49 | 0.14 | |
| 2-Hydroxy-2-phenylethyl | CTL | 8.75 | 1.97 | | CTL | 6.28 | 1.30 | |
| | Egg extract (EE) | 6.24 | 1.03 | | Xcc | 7.57 | 1.55 | |
| | Pieris brassicae | 6.69 | 0.42 | | Pieris | 6.69 | 1.03 | |
| | CTL local | 6.61 | 1.25 | | CTL local | 6.74 | 1.75 | |
| | EE + Pieris brassicae | 6.31 | 0.90 | | Xcc + Pieris | 6.69 | 1.20 | |
| | EE distal | 4.61 | 0.92 | | Xcc distal | 6.93 | 0.43 | |
| | Pieris brassicae distal | 5.82 | 0.95 | | Pieris distal | 7.04 | 1.48 | |
| Gluconasturtin | CTL | 3.92 | 0.82 | | CTL | 2.81 | 0.96 | |
| | Egg extract (EE) | 2.65 | 0.30 | | Xcc | 5.32 | 2.11 | |
| | Pieris brassicae | 5.44 | 2.42 | | Pieris | 9.39 | 1.71 | |
| | CTL local | 3.11 | 0.67 | | CTL local | 3.50 | 1.63 | |
| | EE + Pieris brassicae | 2.28 | 0.79 | | Xcc + Pieris | 2.38 | 1.05 | |
| | EE distal | 2.99 | 0.29 | | Xcc distal | 1.63 | 0.33 | |
| | Pieris brassicae distal | 3.61 | 0.34 | | Pieris distal | 2.92 | 1.81 | |
| Methoxyglucobrassicin | CTL | 0.86 | 0.16 | | CTL | 2.53 | 0.52 | |
| | Egg extract (EE) | 3.23 | 1.47 | | Xcc | 7.55 | 2.04 | |
| | Pieris brassicae | 1.64 | 0.76 | | Pieris | 1.78 | 0.18 | |
| | CTL local | 1.55 | 0.60 | | CTL local | 1.49 | 0.72 | |
| | EE + Pieris brassicae | 3.56 | 0.58 | | Xcc + Pieris | 5.57 | 1.05 | |
| | EE distal | 3.21 | 0.53 | | Xcc distal | 6.59 | 1.30 | |
| | Pieris brassicae distal | 1.14 | 0.12 | | Pieris distal | 4.04 | 0.64 | |
| Neoglucobrassicin | CTL | 0.19 | 0.06 | | CTL | 0.15 | 0.07 | |
| | Egg extract (EE) | 0.09 | 0.05 | | Xcc | 0.14 | 0.05 | |
| | Pieris brassicae | 0.61 | 0.27 | | Pieris | 0.37 | 0.10 | |
| | CTL local | 0.20 | 0.05 | | CTL local | 0.07 | 0.04 | |
| | EE + Pieris brassicae | 0.40 | 0.02 | | Xcc + Pieris | 0.37 | 0.17 | |
| | EE distal | 0.12 | 0.06 | | Xcc distal | 0.14 | 0.06 | |
| | Pieris brassicae distal | 0.35 | 0.03 | | Pieris distal | 0.23 | 0.07 | |
| Total GS | CTL | 1176.43 | 274.90 | | CTL | 632.20 | 39.76 | |
| | Egg extract (EE) | 792.92 | 19.93 | | Xcc | 748.10 | 88.63 | |
| | Pieris brassicae | 1007.54 | 121.95 | | Pieris | 620.10 | 27.03 | |
| | CTL local | 650.25 | 143.47 | | CTL local | 861.80 | 84.44 | |
| | EE + Pieris brassicae | 600.43 | 122.69 | | Xcc + Pieris | 761.98 | 88.80 | |
| | EE distal | 584.07 | 72.98 | | Xcc distal | 755.54 | 140.24 | |
| | Pieris brassicae distal | 583.19 | 109.67 | | Pieris distal | 766.14 | 172.02 | |
