# Supplementary material for: Combined biotic stresses trigger similar transcriptomic responses but contrasting resistance against a chewing herbivore in Brassica nigra
Source: BMC Plant Biol. 2017 Jul 17;17:127. doi: 10.1186/s12870-017-1074-7 (PMC5513356; doi:10.1186/s12870-017-1074-7)
Supplement: Supplementary file 9 — Expression of glucosinolate biosynthesis genes in response to P. brassicae feeding and combined stresses. Values were extracted from microarray data. P. brassicae larvae (orange bars), egg extract/P. brassicae larvae (yellow bars), Xanthomonas campestris pv. raphani/P. brassicae (green bars), Brevicoryne brassicae/P. brassicae (blue bars). Significant differences between single and combined stress are indicated (Student’s t-test, ***P < 0.001, **P < 0.01, *P < 0.05). (PDF 982 kb) [file 12870_2017_1074_MOESM9_ESM.pdf]

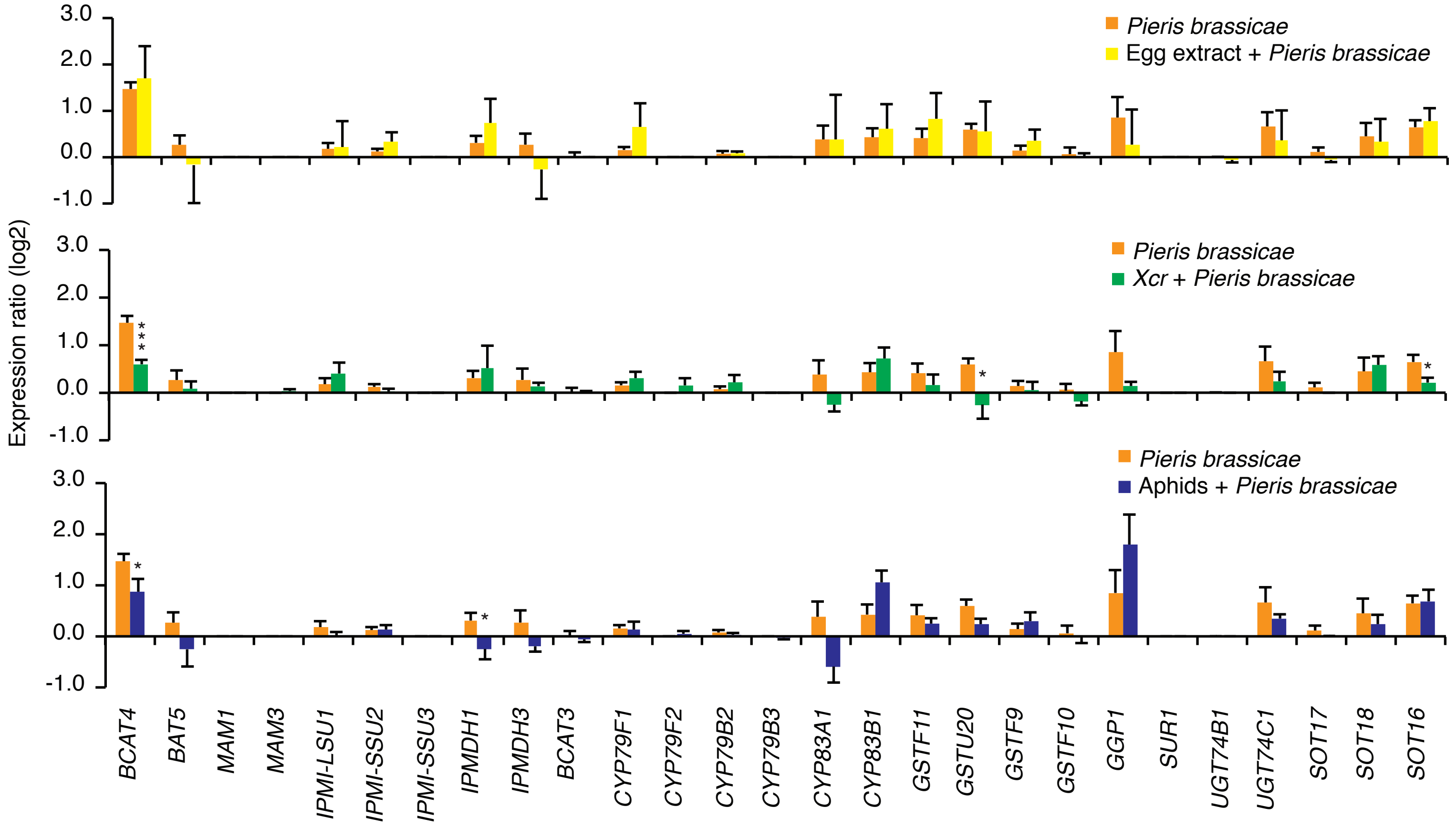

Fig. S6. Expression of glucosinolate biosynthesis genes in response to *P. brassicae* feeding and combined stresses. Values were extracted from microarray data. *P. brassicae* larvae (orange bars), egg extract/*P. brassicae* larvae (yellow bars), *Xanthomonas campestris* pv. *raphani*/*P. brassicae* (green bars), *Brevicoryne brassicae*/*P. brassicae* (blue bars). Significant differences between single and combined stress are indicated (Student's *t*-test, ***$P< 0.001$, **$P< 0.01$, *$P< 0.05$).
