# Supplementary material for: Combined biotic stresses trigger similar transcriptomic responses but contrasting resistance against a chewing herbivore in Brassica nigra
Source: BMC Plant Biol. 2017 Jul 17;17:127. doi: 10.1186/s12870-017-1074-7 (PMC5513356; doi:10.1186/s12870-017-1074-7)
Supplement: Supplementary file 10 — Feeding behavior of P. brassicae larvae in response to combined stresses. Neonate larvae were allowed to feed freely for 2 days (P. brassicae) on 5-week-old B. nigra plants pretreated for 3 days with egg extract (A) or Xanthomonas campestris pv. raphani (B). Representative images from three biological replicates are shown. Scale bar = 1 cm. (PDF 9284 kb) [file 12870_2017_1074_MOESM10_ESM.pdf]

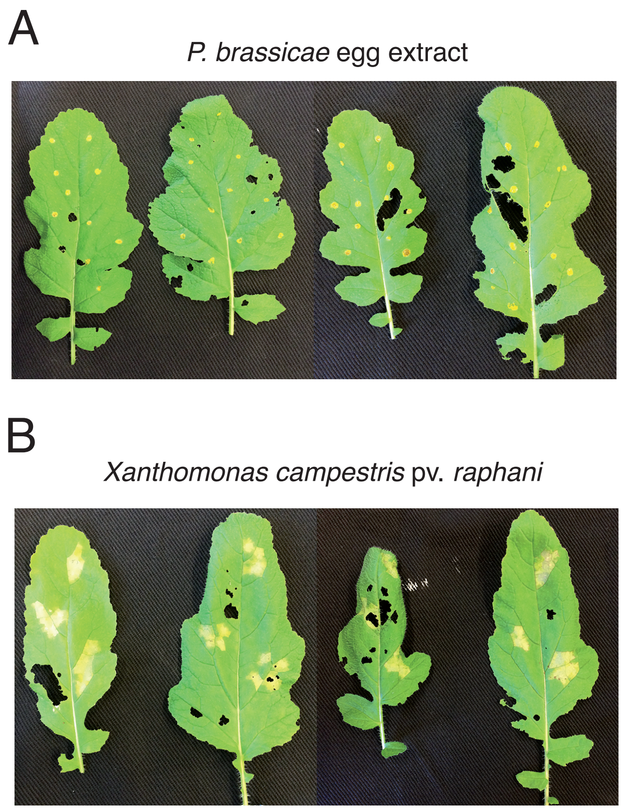

Fig. S7. Feeding behavior of *P. brassicae* larvae in response to combined stresses. Neonate larvae were allowed to feed freely for 2 days (*P. brassicae*) on 5-week-old *B. nigra* plants pretreated for three days with egg extract (A) or *Xanthomonas campestris* pv. *raphani.*
(B). Representative images from three biological replicates are shown. Scale bar = 1 cm.
