# Supplementary material for: Combined biotic stresses trigger similar transcriptomic responses but contrasting resistance against a chewing herbivore in Brassica nigra
Source: BMC Plant Biol. 2017 Jul 17;17:127. doi: 10.1186/s12870-017-1074-7 (PMC5513356; doi:10.1186/s12870-017-1074-7)
Supplement: Supplementary file 11 — Overall experimental design. (PDF 48 kb) [file 12870_2017_1074_MOESM11_ESM.pdf]

Table S4. Overall experimental design

**1st stress (pretreatment)**		**2nd stress (caterpillars)**		**Measurements**	**Figures**
None<br>Egg extract<br>Pathogen	72 h →	None<br>Unconstrained feeding	24 h →	Leaf tissues for microarrays<br>Leaf tissues for JA, SA, GS	**Fig. 1, 2**<br>**Fig. S5**
			7 days →	Caterpillar performance unconstrained	**Fig. 3A**
		Constrained feeding<br>- at site of pretreatment<br>- at site distal to pretreatment	24 h →	Leaf tissues for JA, SA, GS<br>Leaf tissues for QPCR	**Fig. 4**<br>**Fig. 5**
			4 days →	Caterpillar performance constrained	**Fig. 3B**
None<br>Aphids	48 h →	None<br>Unconstrained feeding	24 h →	Leaf tissues for microarrays	**Fig. 1, 2**
