# Supplementary material for: Combined biotic stresses trigger similar transcriptomic responses but contrasting resistance against a chewing herbivore in Brassica nigra
Source: BMC Plant Biol. 2017 Jul 17;17:127. doi: 10.1186/s12870-017-1074-7 (PMC5513356; doi:10.1186/s12870-017-1074-7)
Supplement: Supplementary file 12 — List of primers used for QPCR. (PDF 51 kb) [file 12870_2017_1074_MOESM12_ESM.pdf]

Table S5. List of primers used for QPCR

Gene	Arabidopsis AGI	Primer	Sequence (5' - 3')
BnVSP2	At5g24770	Fwd	GGGAACGTAGCCGAACTCTT
		Rev	CGAAGTCCTTTGGCATAGAAA
BNMYC2	At1g32640	Fwd	GTGGAATCGAGCAAGAGGAA
		Rev	ATCGTTAACCACCGACATACT
BnPR2	At3g57260	Fwd	GTGATAGATTTCTTGGTAAGCA
		Rev	ACCACGATTTCCAACGATCC
BnSAG13	At2g29350	Fwd	AAACTCATGGAAACCGTTTCC
		Rev	TAGATGGATCCAACATTAATATG
BnSAND	At2g28390	Fwd	TGCTTGGAGGGACAGATGC
		Rev	AACCTTGTGTCTGCACATTAG
